# Supplementary material for: The Collaborative IPD of Sleep and Stillbirth (Cribss): is maternal going-to-sleep position a risk factor for late stillbirth and does maternal sleep position interact with fetal vulnerability? An individual participant data meta-analysis study protocol
Source: BMJ Open. 2018 Apr 10;8(4):e020323. doi: 10.1136/bmjopen-2017-020323 (PMC5898330; doi:10.1136/bmjopen-2017-020323)
Supplement: Supplementary file 1 [file bmjopen-2017-020323supp001.pdf]

Appendix 1:

Search strategy for the Collaborative IPD of Sleep and Stillbirth (Cribss) study

Databases or search engines that will be used

A search of the databases: MEDLINE, EMBASE, LILACS, Web of Science, OpenGrey, and Google Scholar, will be conducted, for the purpose of locating published research about an association between maternal sleep position and late pregnancy stillbirth. We will also access WHO International Clinical Trials Registry Platform to identify any ongoing and registered trials. Proceedings from International Stillbirth Alliance (ISA) annual conferences and The International Society for the Study and Prevention of Perinatal and Infant Death (ISPID) international conferences will be manually searched. Published perinatal conference abstracts will be identified through the above database searches. Experts in the field and the collaborative group will be asked for their knowledge of any unpublished studies.

Limits applied

To increase the likelihood of identifying all relevant studies, the reference lists of all retrieved articles will be hand searched. No language restriction will be applied.

List the search terms used

Three search terms will be used to search the databases with the article title, abstracts and body all searched. The search terms are:

- stillbirth
- fetal death
- sleep

and synonyms. The search terms will be tested to check that they effectively located the types of articles that are consistent with the inclusion criteria prior to conducting the search in all engines.

Document the search process

The following search was conducted sequentially using the search terms in MEDLINE on 20th November 2016.

Search engine		Search terms	# Retrieved:
**MEDLINE**			
MEDLINE	1	Stillbirth/	3851
MEDLINE	2	(stillbirth* or still-birth* or stillborn* or still-born*).ti,ab,kf.	13691
MEDLINE	3	Fetal Death/	24585
MEDLINE	4	((fetal or foetal or fetus or foetus) adj death*).ti,ab,kf.	8769
MEDLINE	5	((fetal or foetal or fetus or foetus) adj3 (loss or losses)).ti,ab,kf.	4804
MEDLINE	6	Perinatal Death/	860
MEDLINE	7	((perinatal or peri-natal) adj death*).ti,ab,kf.	4007
MEDLINE	8	((prenatal or pre-natal or intrauterine or intra-uterine or antepartum or ante-partum or antenatal or ante-natal) adj death*).ti,ab,kf.	2026
MEDLINE	9	or/1-8	46353
MEDLINE	10	Sleep/	46957
MEDLINE	11	((sleep or sleeping) adj (position* or practice* or posture*)).ti,ab,kf.	1354
MEDLINE	12	maternal sleep*.ti,ab,kf.	139
MEDLINE	13	or/10-12	47711
MEDLINE	14	9 and 13	23
