# Supplementary material for: The Collaborative IPD of Sleep and Stillbirth (Cribss): is maternal going-to-sleep position a risk factor for late stillbirth and does maternal sleep position interact with fetal vulnerability? An individual participant data meta-analysis study protocol
Source: BMJ Open. 2018 Apr 10;8(4):e020323. doi: 10.1136/bmjopen-2017-020323 (PMC5898330; doi:10.1136/bmjopen-2017-020323)
Supplement: Supplementary file 2 [file bmjopen-2017-020323supp002.pdf]

Collaborative IPD Analysis of Sleep and Stillbirth

Statistical Analysis Plan Version 2

Project Title An individual participant data meta-analysis of going-to-sleep position and risk of late pregnancy stillbirth

Field of research 11402 Obstetrics and Gynaecology

Date of first version	17/08/2017
Last updated	28/01/2018

Table of content

1. INTRODUCTION AND OVERVIEW 1

1.1. Study overview 1

1.2. Objectives 1

1.3. Eligibility criteria 1

1.4. Definition of Primary Outcome 2

1.5. Definition of Secondary Outcome 3

1.6. Definition of Primary Exposure 3

1.7. Definition of Other Variables 4

1.7.1. Potential confounders for main questions 4

1.7.2. Additional variables for secondary questions 7

2. STATISTICAL ANALYSIS PLAN 8

2.1. Analysis population 8

2.2. Sample size 8

2.3. Descriptive statistics 8

2.4. Analysis for main questions 8

2.5. Analysis for secondary questions 9

REFERENCES 10

1. Introduction and Overview

1.1. Study overview

Accumulating evidence has shown an association between maternal supine sleep position and stillbirth in late pregnancy. Advising women not to go to sleep on their back can potentially reduce late stillbirth rate by 3.7%-10% [1-3]. However, the association between maternal right side sleep position and stillbirth is inconsistent across studies. Furthermore, individual studies are underpowered to investigate interactions between maternal sleep position and fetal vulnerability, which is potentially important for producing clear and tailored public health messages on safe going-to-sleep position. We aim to use individual participant data (IPD) from existing studies to assess whether right-side and supine going-to-sleep positions are independent risk factors for late stillbirth and test the interaction between sleep position and indicators of fetal vulnerability.

1.2. Objectives

The main questions to be addressed:

1. Is maternal going-to-sleep position associated with late stillbirth?
2. Are indicators of fetal vulnerability, including: maternal obesity, SGA, maternal smoking, maternal second-hand tobacco exposure, substance use, alcohol consumption, maternal medical conditions (including pre-existing hypertension and diabetes), and maternal perception of fetal movements associated with late stillbirth, and does maternal going-to-sleep position interact with indicators of fetal vulnerability to influence the risk of late stillbirth? Does birthweight centile interact with maternal going-to-sleep position to influence the risk of late stillbirth?

Secondary questions to be addressed by the first cycle of Cribss IPD meta-analysis are:

1. Is sleep disturbed breathing associated with late stillbirth? Is (are) going-to-sleep position(s) associated with greater risk of late stillbirth in women with sleep

disturbed breathing? Is sleep disturbed breathing a moderator for sleep position in relation to late stillbirth?

2. Are factors that may influence vena caval compression (eg, long sleep duration, sleeping during the day, restless legs,) associated with risk of late stillbirth? Do these factors interact with going-to-sleep position?
3. Do women who report they received advice about sleep position have lower risk of late stillbirth compared with women who did not receive such advice?
4. Do women who report they received advice about awareness of fetal movements have a lower risk of late stillbirth than women who did not receive such advice?

1.3. Eligibility criteria

Study inclusion criteria (regardless of whether the study is published or unpublished):

1. Case-control and prospective cohort studies which collected:
    - Maternal going-to-sleep position during pregnancy and
    - Pregnancy outcome that included stillbirth and
    - Aimed to recruit controls with an on-going pregnancy at similar gestation to the cases
2. Randomised controlled trials which collected:
    - Maternal going-to-sleep position during pregnancy and
    - Pregnancy outcome data that included stillbirth

Participant level exclusion criteria:

- Multiple pregnancy in the third trimester
- Major congenital abnormality at study entry or major congenital abnormality as a cause of death found post study entry or post-randomisation in randomised controlled trials

- Gestation less than 28 weeks when last sleep position data during pregnancy was collected
- Termination of pregnancy at greater than or equal to 28 weeks
- Received study intervention that might have an impact on going-to-sleep position.

1.4. Definition of Primary Outcome

Late stillbirth

We will use the definition recommended by World Health Organisation (WHO) for international comparison: "a baby born with no signs of life at or after 28 weeks' gestation"[4]. Intrapartum stillbirth will be included in the analysis with the rationale that supine going-to-sleep position may result in a vulnerable baby that is unable to tolerate labour.

1.5. Definition of Secondary Outcome

Small for Gestational Age (SGA)

SGA will be defined using (1) the definition in each study, (2) Customised centiles [5, 6], (3) WHO or uniform population standards, and (4) INTERGROWTH-21st. For primary analysis, SGA is defined as birthweight less than the 10th customized birth centile. Mother estimated date of stillbirth (before or on the same day of the diagnosis of stillbirth) will be used to calculate the gestation for SGA. If estimated date of stillbirth is unavailable, date of diagnosis of stillbirth will be used. If date of diagnosis is unavailable, baby date of birth will be used.

1.6. Definition of Primary Exposure

Maternal going-to-sleep position

Going-to-sleep position for the primary analysis is the information collected closest to time of interview on going-to-sleep position but within the 'last two weeks' prior to interview in controls and before stillbirth in cases. It has been shown in previous studies, that last night going-to-sleep position is highly correlated to the usual going-to-sleep position within the last two weeks. Going-to-sleep position will be categorised as left side, right side, variable sides, supine, prone, and propped up. Left side will be used as the reference group during

the analysis. Depending on the similarity of the risk estimates, going-to-sleep position may be further merged to fewer groups such as supine vs non-supine groups in the analysis of interaction.

1.7. Definition of Other Variables

1.7.1. Potential confounders for main questions

Maternal age

Maternal age is defined as the age at the time of interview for controls and the age at the time of stillbirth for cases. Maternal age should be calculated by date of birth where possible. Self-reported age at the interview will be used if maternal date of birth or date of interview is unavailable. The relationship between maternal age and stillbirth will be explored using a generalized additive model (GAM). Depending on the linearity of the relationship, maternal age will be further explored as either a continuous variable or a categorical variable.

Maternal ethnicity

Maternal ethnicity is defined as self-reported ethnicity. Maternal ethnicity will be assessed 1) per original study protocol, eg: using prioritization in New Zealand studies [7]. 2) by IPD agreed standardization rule and will be categorised as White (including NZ and Australia European, British, Irish and Gypsy, and other Europeans), Black (including British Black, African, and Caribbean), South Asian (including Indian, Pakistani, Bangladeshi, Sri Lankan, Nepali, Bhutanese, Afghan and Maldivian), South East and East Asian (including Chinese, Japanese, Korean, Vietnamese, Malaysian, and Indonesian), Maori, Pacific Islanders, and others. If the number in some pre-defined group is insufficient for analysis, ethnicity may be further aggregated into fewer groups such as white and non-white.

Maternal parity

Parity is defined as number of previous births after 24 weeks gestation. Maternal parity will be assessed by IPD agreed standardization rule (births after 24 weeks gestation). Maternal parity will be initially explored as six groups: 0, 1, 2, 3, 4, and 5 or more. Depending on the similarity of the risk estimate, maternal parity will be further merged to fewer groups where appropriate.

Maternal education level

Maternal education level will be used as a surrogate for social deprivation and is defined as the highest education the participant has completed at the time of interview. Maternal education level will be explored as five groups: 1. Primary; 2. Secondary; 3. Non-university trade education (vocational training); 4. University; 5. Post-graduate degree. Depending on the similarity of the risk estimates, maternal education level will be further merged to fewer groups where appropriate.

Marital status

Marital status will be categorised as single (including never married, divorced, widowed and separated), co-habiting (including de facto) or married (including civil partnership). Depending on the data availability, marital status can be also categorised as single or in relationship (cohabiting and married).

Maternal BMI

Maternal BMI is defined as the earliest collected weight during pregnancy or before pregnancy (kg) divided by squared maternal height (m). Where maternal weight or maternal height is unavailable, the earliest BMI recorded during or before pregnancy will be used. The proportion of participants with their earliest weight measurement recorded during the first trimester will be calculated.

The relationship between maternal BMI and stillbirth (eg: any dose –response relation) will be explored using GAM. Depending on the linearity of the relationship, maternal age will be further explored as either a continuous variable or a categorical variable.

Maternal obesity

Maternal earliest BMI during pregnancy equal to or greater than 30 is considered as obese. Maternal obesity will be explored as an indication of fetal vulnerability.

Maternal smoking

Maternal smoking status will be explored as three groups: current smoker (including those who stopped in 2nd, or 3rd trimester), used to smoke but stopped within the 1st trimester, and never smoked. Depending on the similarity of the risk estimate, maternal smoking status may be merged to two groups in the final analysis.

Environmental tobacco exposure

Environmental tobacco exposure is defined as living with a smoker (including partner or any other person living in the same household) anytime during pregnancy.

Substance use

Maternal substance use will be defined as: 1) any use of recreational drugs during any stage in pregnancy, 2) marijuana use in the first three months during pregnancy, 3) marijuana use in the last month during pregnancy, and 4) marijuana in the last week before interview / stillbirth.

Alcohol consumption

Maternal alcohol exposure will be assessed by the largest number of standard drinks on one occasion during any phase of pregnancy. Maternal alcohol exposure will be also assessed by the average standard drinks per week in the month before interview for controls and before stillbirth for cases. Average standard drinks per week will be coded as 'less than 1', '1 to 2', '3 to 4', and '5 or more'. Depending on the similarity of the risk estimates, maternal alcohol exposure may be merged to fewer groups in the final analysis.

Maternal medical conditions (eg: diabetes, hypertension)

Maternal medical conditions will be defined as per original study using local clinical diagnoses as most of the information required to standardise diagnostic criteria across studies is unavailable. However, local diagnostic criteria will be compared if any medical condition is found to be an effect modifier.

Maternal perception of fetal movements

Maternal perception of fetal movements will use changes in fetal movement frequency within the last two weeks for the main questions. Changes in fetal movement frequency will be defined as 1) increased, 2) decreased and 3) same. Particularly, unknown changes will be categorised as same.

Getting up to go to toilet

This is defined as maternal self-reported number of times getting up to go to the toilet on the last night, or if last night information is unavailable, self-reported average over the 'last two weeks' prior to interview in controls and before stillbirth in cases.

Sleep duration

This is defined as self-estimated sleep duration for the last night, or if last night information is unavailable, self-reported average over the 'last two weeks' prior to interview in controls and before stillbirth in cases.

Daytime napping

Daytime napping is defined as self-reported frequency of sleep during day time per week in the most recent available time frame during pregnancy.

Birthweight centile

Birthweight centile will be calculated using (1) the definition in each study, (2) Customised centiles [5, 6], (3) WHO or uniform population standards, and (4) INTERGROWTH-21st.

1.7.2. Additional variables for secondary questions

Sleep disturbed breathing

Questions adopted from the Berlin questionnaire for obstructive sleep apnea will be used to investigate sleep disturbed breathing in this study [8]. The following information will be evaluated: (1) awareness of snoring or not, (2) if the snoring bothering other people, (3) snoring volume, (4) stopping breathing during the sleep, (5) coughing or choking during sleep, (6) daytime sleepiness, (7) the likelihood of doing off at various occasions including sitting while reading, watching TV, sitting in public, sitting in a car, lying down for rest in the afternoon, sitting while talking, after lunch, and in a car when stopping at the traffic light.

Restless legs syndrome

Restless legs syndrome is defined as regular jerking movements of arms or legs during sleep during any phase of the pregnancy.

Advice about sleep position

Advice about sleep position will be assessed as binary data- received advice on sleep position or not during pregnancy. The sources and the content of the advice will be evaluated further according to the data availability.

Advice about fetal movement

Advice about fetal movement will be assessed as binary data: 1)received advice on fetal movement 2) did not receive advice or 3) cannot recall advice during pregnancy. The sources and the content of the advice will be evaluated further according to the data availability.

2. Statistical Analysis Plan

2.1. Analysis population

Participants who meet the above inclusion criteria (see 1.3) will be included in the analysis. Participants with missing data for variables to be included in multivariable model will not have those data-points imputed.

2.2. Sample size

We anticipate that 700 cases and 1800 controls will be included in the primary analysis. This sample size will have 80% power to detect an odds ratio of 1.86 for a factor with 3% prevalence in controls, and an odds ratio of 1.47 for a factor with 10% prevalence in controls, and an odds ratio of 1.35 for a factor with 20% prevalence in controls.

2.3. Descriptive statistics

Descriptive statistics for exposure and confounders will be presented in tables by cases and controls. All data will be explored for missing data and checked for distribution.

Continuous variables: GAM will be used to explore their relationship between continuous variables and the main outcome (late stillbirth). If the relationship is linear, the variable will be presented as mean and standard deviation, or median and quartiles when considered appropriate, and will be fitted in the logistic model as continuous variables. If the relationship is not linear, the variable will be categorised or fitted with non-linear terms.

Categorical variables: categorical variables will be described in terms of prevalence (frequency) and percentages of the number of participants examined.

2.4. Analysis for main questions

An individual participant data (IPD) analysis will be performed. A one stage approach to analysis will be taken so that the individual participant data from all eligible studies are included in a single model. Logistic regression models will be used for the binary outcome

(late stillbirth). A fixed study effect and a study site effect will be included in the model specification as strata. Univariable analysis will be performed to evaluate the association between sleep position and late stillbirth risk. The interaction between sleep position and factors indicating a vulnerable pregnancy will be assessed in bi-variable models. A multivariable model will be developed incorporating previously reported confounders and any significant interaction terms, once it has been established what cofounders can be controlled for consistently across studies. Estimate of risk will be reported as odds ratio and 95% confidence intervals.

Statistical analyses will be performed using SAS (SAS Institute Inc., Cary NC USA). If an important confounder is not available for one or more studies, sensitivity analysis will be conducted, with and without these studies, to compare risk estimates. For missing data in each individual study, no imputation will be carried out.

2.5. Analysis for secondary questions

Secondary questions investigating other exposures and the risk of stillbirth will be first analysed by each study, and then the merged data set will be analysed through a one stage approach. The rationale to first explore each study independently is because these questions have never been explored before and it is important to see if there is any exposure outcome effect in each individual study. Logistic regression models will be used for the binary outcome (late stillbirth). A fixed study effect and a study site effect will be included in models as strata. Univariable analysis will be performed to evaluate the association between exposures (data indicating sleep disturbed breathing, indications of compression of vena cava, and advice of sleep position and fetal movement) and late stillbirth risk. The interaction between secondary exposures and sleep position will be explored in bi-variable models. A multivariable model will be developed incorporating appropriate confounders/interaction. If an important confounder is not available for one or more studies, sensitivity analysis will be conducted, with and without these studies, to compare risk estimates. Estimate of risk will be reported as odds ratio and 95% confidence intervals.

Statistical analyses will be performed using SAS (SAS Institute Inc., Cary NC USA).

References

1. McCowan LME, Thompson JMD, Cronin RS, Li M, Stacey T, Stone PR, et al. Going to sleep in the supine position is a modifiable risk factor for late pregnancy stillbirth; Findings from the New Zealand multicentre stillbirth case-control study. PloS one. 2017;12(6):e0179396.
2. Platts J, Mitchell EA, Stacey T, Martin BL, Roberts D, McCowan L, et al. The Midland and North of England Stillbirth Study (MiNESS). BMC pregnancy and childbirth. 2014;14:171.
3. Gordon A, Raynes-Greenow C, Bond D, Morris J, Rawlinson W, Jeffery H. Sleep position, fetal growth restriction, and late-pregnancy stillbirth: the Sydney stillbirth study. Obstetrics and gynecology. 2015 Feb;125(2):347-55.
4. WHO. Maternal, newborn, child and adolescent health: Data, statistics and epidemiology: WHO; [cited 2016 21 November 2016]. Available from: http://www.who.int/maternal_child_adolescent/epidemiology/stillbirth/en/.
5. Gardosi J, Chang A, Kalyan B, Sahota D, Symonds EM. Customised antenatal growth charts. Lancet. 1992 Feb 01;339(8788):283-7.
6. McCowan L, Stewart AW, Francis A, Gardosi J. A customised birthweight centile calculator developed for a New Zealand population. The Australian & New Zealand journal of obstetrics & gynaecology. 2004 Oct;44(5):428-31.
7. Minitry of Health. Ethnicity Data Protocols for the Health and Disability Sector. Wellington: Ministry of Health2004. Available from: http://www.health.govt.nz/publication/ethnicity-data-protocols-health-and-disability-sector
8. Netzer NC, Stoohs RA, Netzer CM, Clark K, Strohl KP. Using the Berlin Questionnaire to identify patients at risk for the sleep apnea syndrome. Annals of internal medicine. 1999 Oct 05;131(7):485-91.
