# Supplementary material for: The Collaborative IPD of Sleep and Stillbirth (Cribss): is maternal going-to-sleep position a risk factor for late stillbirth and does maternal sleep position interact with fetal vulnerability? An individual participant data meta-analysis study protocol
Source: BMJ Open. 2018 Apr 10;8(4):e020323. doi: 10.1136/bmjopen-2017-020323 (PMC5898330; doi:10.1136/bmjopen-2017-020323)
Supplement: Author's manuscript [file bmjopen-2017-020323.draft_revisions.pdf]

BMJ Open is committed to open peer review. As part of this commitment we make the peer review history of every article we publish publicly available.

When an article is published we post the peer reviewers' comments and the authors' responses online. We also post the versions of the paper that were used during peer review. These are the versions that the peer review comments apply to.

The versions of the paper that follow are the versions that were submitted during the peer review process. They are not the versions of record or the final published versions. They should not be cited or distributed as the published version of this manuscript.

BMJ Open is an open access journal and the full, final, typeset and author-corrected version of record of the manuscript is available on our site with no access controls, subscription charges or pay-per-view fees (http://bmjopen.bmj.com).

If you have any questions on BMJ Open's open peer review process please email info.bmjopen@bmj.com

BMJ Open

The Collaborative IPD of Sleep and Stillbirth (Cribss) - an Individual Participant Data Meta-Analysis study protocol

Journal:	*BMJ Open*
Manuscript ID	bmjopen-2017-020323
Article Type:	Protocol
Date Submitted by the Author:	28-Oct-2017

Complete List of Authors:	Li, Minglan; University of Auckland, Department of Obstetrics and Gynaecology<br>Thompron, John; University of Auckland, Paediatrics: Child and Youth Health<br>Cronin, Robin; University of Auckland, Department of Obstetrics and Gynaecology<br>Gordon, Adrienne; University of Sydney - Camden Campus, Department of Paediatrics<br>Raynes-Greenow, Camille; The University of Sydney, School of Public Health<br>Heazell, Alexander; University of Manchester, Maternal and Fetal Health Research Centre<br>Stacey, Tomasina; University of Leeds, School of Healthcare<br>Culling, Vicki ; Vicki Culling Associates<br>Bowring, Victoria; Stillbirth Foundation Australia<br>Mitchell, Edwin; University of Auckland, Paediatrics<br>McCowan, Lesley; University of Auckland<br>Askie, Lisa; University of Sydney, National Health and Medical Research Council Clinical Trials Centre

Keywords:	Stillbirth, Sleep position, Individual participant data meta-analysis, Small for gestational age, Fetal movement

SCHOLARONE™
Manuscripts

The Collaborative IPD of Sleep and Stillbirth (Cribss) - an Individual Participant Data Meta-Analysis study protocol

Minglan Li[1] m.li@auckland.ac.nz

John MD Thompson[1,2] j.thompson@auckland.ac.nz

Robin S Cronin[1] r.cronin@auckland.ac.nz

Adrienne Gordon[3,4] adrienne.gordon@sydney.edu.au

Camille Raynes-Greenow[5] camille.raynes-greenow@sydney.edu.au

Alexander E P Heazell[6,7] alexander.heazell@manchester.ac.uk

Tomasina Stacey[8] t.stacey@leeds.ac.uk

Vicki Culling[9] vicki@vca.co.nz

Victoria Bowring[10] victoria@stillbirthfoundation.org.au

Edwin A Mitchell[2] e.mitchell@auckland.ac.nz

Lesley ME McCowan[1] l.mccowan@auckland.ac.nz

Lisa Askie[11] lisa.askie@ctc.usyd.edu.au

1. Department of Obstetrics and Gynaecology, University of Auckland, Auckland, New Zealand. 2. Department of Paediatrics and Child Health, University of Auckland, Auckland, New Zealand. 3. Department of Newborn Care, Royal Prince Alfred Hospital Women and Babies, Sydney, Australia. 4. The University of Sydney, Charles Perkins Centre, University of Sydney, Sydney, NSW, Australia. 5. The University of Sydney, Sydney School of Public Health, Sydney, NSW, Australia. 6. Maternal and Fetal Health Research Centre, Division of Developmental Biomedicine, Faculty of Medical and Human Sciences, University of Manchester, UK. 7. St. Mary's Hospital, Central Manchester University Hospitals NHS Foundation Trust, Manchester Academic Health Science Centre, Manchester, UK. 8. School of Healthcare, University of Leeds, Leeds, UK. 9. Vicki Culling Associates, Auckland, New

For peer review only - http://bmjopen.bmj.com/site/about/guidelines.xhtml

Zealand. 10. Stillbirth Foundation, Australia. 11. National Health and Medical Research Council Clinical Trials Centre, University of Sydney, Camperdown, Australia.

Co-responding author:

Minglan Li

Department of Obstetrics and Gynaecology,

University of Auckland,

Private Bag 92019

Auckland, New Zealand

For peer review only

For peer review only - http://bmjopen.bmj.com/site/about/guidelines.xhtml

ABSTRACT

Introduction: Accumulating evidence has shown an association between maternal supine going-to-sleep position and stillbirth in late pregnancy. Advising women not to go to sleep on their back can potentially reduce late stillbirth rate by 9%. However, the association between maternal right-sided going-to-sleep position and stillbirth is inconsistent across studies. Furthermore, individual studies are underpowered to investigate interactions between maternal going-to-sleep position and fetal vulnerability, which is potentially important for producing clear and tailored public health messages on safe going-to-sleep position. We will use individual participant data (IPD) from existing studies to assess whether right-side and supine going-to-sleep positions are independent risk factors for late stillbirth and test the interaction between going-to-sleep position and fetal vulnerability.

Methods and Analysis: An IPD meta-analysis approach will be utilised using the Cochrane Collaboration-endorsed methodology. We will identify case-control and prospective cohort studies and randomised trials which collected maternal going-to-sleep position data and pregnancy outcome data that included stillbirth. The primary outcome is stillbirth. A one stage procedure meta-analysis, stratified by study with adjustment of a priori confounders will be carried out.

Ethics and dissemination: The IPD meta-analysis has obtained central ethics approval from the New Zealand Health and Disability Ethics Committee, ref: NTX/06/05/054/AM06. Individual studies should also have ethical approval from relevant local ethics committees. Interpretation of the results will be discussed with consumer representatives. Results of the study will be published in peer-reviewed journals and presented at international conferences.

Systematic review registration: PROSPERO registration number: CRD42017047703

STRENGTHS AND LIMITATIONS OF THIS STUDY

- Late stillbirth is a rare event in high-income countries, and individual participant data meta-analysis of several studies can yield a sufficiently large sample size for exploring interactions and subgroup analysis that are difficult to undertake within a single study.

For peer review only

For peer review only - http://bmjopen.bmj.com/site/about/guidelines.xhtml

- There is no restriction on language or countries where the study was conducted, therefore the results from this study are likely to be generalisable.
- It is the first IPD meta-analysis examining the association between maternal going-to-sleep position in late pregnancy and the risk of stillbirth, and the potential interactions with other stillbirth risk factors. The results from this study are likely to contribute important messages for a public health intervention.
- Service users will oversee the conduct of the study. Their involvement will help to design appropriate research questions and will help the implementation and translation of the research outcomes.
- One limitation of the study is that the maternal going-to-sleep positions are likely to be self-reported.

For peer review only

For peer review only - http://bmjopen.bmj.com/site/about/guidelines.xhtml

INTRODUCTION

Stillbirth, the death of a baby before birth, is a major global burden affecting more than 2.6 million families per year [1]. In high-income countries, the rate of late stillbirth (28 weeks or greater) varies widely from 1.3 to 8.8 per 1,000 births [2] and is approximately twice as common as neonatal death [3]. Importantly, the annual rate of reduction for neonatal death is twice that of stillbirth [2]. The variations between countries suggest it is possible to further reduce late stillbirth. Importantly, maternal characteristics present in early pregnancy only explain a small amount of the risk for late stillbirth [4]. Therefore, significant reductions in late stillbirth require identification of additional maternal risk factors amenable to modification during pregnancy [5].

Accumulating evidence suggests that supine going-to-sleep position may be a modifiable risk factor for stillbirth in late pregnancy. Stacey *et al.* first reported an association between going-to-sleep position and late stillbirth, with women who did not go-to-sleep on their left side, the night before the baby was suspected to have died, having an increased odds of stillbirth [6]. Among non-left sided sleepers, the odds were greater in women who went to sleep supine; and there was also a borderline increase in odds in women who went to sleep on their right side [6]. Similar associations between supine going-to-sleep position and late stillbirth have since been reported by several studies [7-9]. In addition to the epidemiologic evidence, a number of physiological studies have suggested that the relationship between supine going-to-sleep position and late stillbirth is biologically plausible. Significant hemodynamic changes in maternal and fetal circulation have been observed in relation to maternal position in late pregnancy, with decreased maternal cardiac output and uterine blood flow [10], and pulsatility index in the fetal middle cerebral artery (a surrogate for fetal hypoxia) [11] seen in maternal supine position when compared to left position. A recent study by Stone *et al*. has shown that when the mother is in the supine position, the fetus spends more time in behavioural state 1 (fetal quiescence) and less time in active fetal behavioural state 4, compared to when the mother is on her left side, indicating supine

For peer review only

For peer review only - http://bmjopen.bmj.com/site/about/guidelines.xhtml

position may be a mild hypoxic stressor [12]. It was hypothesised that these physiological changes associated with supine position are related to the direct compression of the inferior vena cava by the gravid uterus [13]. Furthermore, supine sleep position is also associated with sleep disturbed breathing and obstructive sleep apnoea [14], which have also been associated with pregnancy complications such as pre-eclampsia, fetal growth restriction [15], and gestational diabetes [15, 16]. These pregnancy complications are known risk factors for stillbirth [17], and might represent another mechanism that contributes to the association between supine going-to-sleep position and late stillbirth.

The findings from the epidemiological studies combined with the supportive physiological evidence suggest that the association between supine sleep position and late stillbirth is likely to be causal. Informing pregnant women and their healthcare providers about optimal going-to-sleep position in late pregnancy is a strategy that may reduce stillbirth and is potentially harmless. Therefore, there is an urgent need to assess the accumulated evidence to develop a public health campaign. However, there are some unanswered questions that are critical for developing clear public health messages. Firstly, it is unclear whether right sided going-to-sleep position is a risk factor for late stillbirth. A borderline increase in risk was reported with right side compared to left side going-to-sleep position in the Stacey *et al.* study. However, this association was not found in other studies [7, 9]. The inconsistent finding of right side going-to-sleep position warrants further clarification so that clear advice about whether women should be advised to go-to-sleep on either side or only on their left side can be developed. Secondly, there is no evidence whether there are groups of women who are at elevated risk when they go-to-sleep in a suboptimal position (such as those who smoke, are overweight or have small babies etc.) and how other stillbirth risk factors interact with sleep position. Stillbirth is the end point of diverse pathological processes. Multiple risk factors and pathological events can contribute at different time points and cumulatively lead to the final event. Our research group has hypothesised a triple-risk framework for late stillbirth that cannot be explained by one risk factor or condition alone [18]. We speculate

For peer review only - http://bmjopen.bmj.com/site/about/guidelines.xhtml

that three groups of factors namely maternal factors (eg, obesity, smoking), fetal and placental factors (eg, a small for gestational age (SGA) fetus) and an additional stressor(s) (eg, reduced uterine blood flow associated with supine position) in themselves may be insufficient to cause the death, but their combination may have a lethal effect [18]. Individual stillbirth case control studies published to date have insufficient power to explore fully the interactions between supine going-to-sleep position, markers of fetal vulnerability and adverse maternal factors. Furthermore, it is important to explore other factors that may also be associated with supine sleep position such as SGA, reduced fetal movements and sleep disturbed breathing, as this may provide insights into the potential mechanism of risk associated with the supine position.

The Collaborative IPD Sleep and Stillbirth (Cribss) group was established in December 2016. We aim to synthesise the current evidence about going-to-sleep position and stillbirth risk. Additionally we will address the above unanswered questions by combining and analysing the individual participant data from all available studies in an individual participant data (IPD) meta-analysis. IPD meta-analysis is considered the gold standard approach to evidence synthesis as it has the potential to improve the precision and reliability of the results obtained from individual studies [19]. In contrast to the traditional approach of meta-analysis, which extracts summary (aggregate) data from study publications, an IPD meta-analysis uses line-by-line original data sourced directly from the researchers responsible for the relevant studies. An IPD meta-analysis involves the central collection, checking, harmonisation and re-analysis of the original data of all eligible participants from each of the available studies. With proper quality assessment and standardisation processes, an IPD meta-analysis can model complex relationships, which traditional meta-analyses are not able to do [20]. It is particularly useful in evaluating multi-factorial frameworks by evaluating critical outcome determinants and their interactions.

For peer review only - http://bmjopen.bmj.com/site/about/guidelines.xhtml

OBJECTIVES

The main questions to be addressed by the Cribss IPD meta-analysis are:

1. Is maternal going-to-sleep position associated with late stillbirth?
2. Are indicators of fetal vulnerability, including: maternal obesity, SGA, maternal smoking, maternal second-hand tobacco exposure, substance use, alcohol consumption, maternal medical conditions (including pre-existing hypertension and diabetes), and maternal perception of fetal movements associated with late stillbirth?
3. Does maternal going-to-sleep position interact with indicators of fetal vulnerability to influence the risk of late stillbirth?

Secondary questions to be addressed by the first cycle of Cribss IPD meta-analysis are:

1. Is sleep disturbed breathing associated with late stillbirth? Is (are) going-to-sleep position(s) associated with greater risk of late stillbirth in women with sleep disturbed breathing?
2. Are factors that may influence vena caval compression (eg, long sleep duration, sleeping during the day, restless legs,) associated with risk of late stillbirth? Do these factors interact with going-to-sleep position?
3. Do women who report they received advice about sleep position have lower risk of late stillbirth compared with women who did not receive such advice?
4. Do women who report they received advice about awareness of fetal movements have a lower risk of late stillbirth than women who did not receive such advice?

METHODS AND ANALYSIS

This study will apply an IPD meta-analysis approach, and will follow the methodology endorsed by the Cochrane Collaboration where applicable [21, 22]. We will adhere to the Preferred Reporting Items for Systematic Reviews and Meta-analysis (PRISMA) IPD statement for reporting findings. The study will be conducted by the Collaborative IPD Sleep and Stillbirth (Cribss) group which comprises the participating study investigators, an IPD expert, and consumer representatives. The coordination centre is located in the department

For peer review only

For peer review only - http://bmjopen.bmj.com/site/about/guidelines.xhtml

of Obstetrics and Gynaecology at the University of Auckland, Auckland, New Zealand. We have registered the IPD Study with the PROSPERO international prospective register of systematic reviews (CRD42017047703).

Eligibility criteria

Study inclusion criteria (regardless of whether the study is published or unpublished):

1. Case-control and prospective cohort studies which collected:
    - Maternal going-to-sleep position during pregnancy and
    - Pregnancy outcome that included stillbirth and
    - Aimed to recruit controls with an on-going pregnancy at similar gestation to the cases
2. Randomised controlled trials which collected:
    - Maternal going-to-sleep position during pregnancy and
    - Pregnancy outcome data that included stillbirth and
    - Did not test an intervention that might have an impact on going-to-sleep position

Participant level exclusion criteria:

- Multiple pregnancy in the third trimester
- Major congenital abnormality at study entry or major congenital abnormality as a cause of death found post study entry or post-randomisation in randomised controlled trials
- Gestation less than 28 weeks when last sleep position data during pregnancy was collected
- Termination of pregnancy at greater than or equal to 28 weeks

Information sources and search strategy

We will develop the search strategy according to the Cochrane Collaboration guidelines prior to the initial literature search. A search of the databases: MEDLINE, EMBASE, LILACS, Web of Science, OpenGrey, and Google Scholar, will be conducted, for the purpose of locating

For peer review only

For peer review only - http://bmjopen.bmj.com/site/about/guidelines.xhtml

published research about an association between maternal sleep position and late pregnancy stillbirth. We will also access the WHO International Clinical Trials Registry Platform to identify any ongoing and registered trials. Proceedings from International Stillbirth Alliance (ISA) annual conferences and The International Society for the Study and Prevention of Perinatal and Infant Death (ISPID) international conferences. Published perinatal conference abstracts will also be identified through the above database searches. Experts in the field and the collaborative group will be asked about their knowledge of any unpublished studies. To increase the likelihood of identifying all relevant studies, the reference lists of all retrieved articles will be hand searched. No language restriction will be applied.

Four search terms will be used to search the databases with the article title, abstracts and body all searched. The search terms are: 'stillbirth', 'fetal death', 'perinatal death' and 'sleep' and synonyms. The search terms will be tested to check that they effectively located the types of articles that are consistent with the inclusion criteria prior to conducting the search in all engines. An example of a detailed MEDLINE search strategy is presented in supplementary appendix 1.

Selection process

Study eligibility will be assessed independently by two members of the Cribss group, any disagreements will be adjudicated by a third member. Eligibility assessment will be based on published protocols, method sections from publications, and unpublished protocols and, or study information requested from potential eligible study investigators. All potential eligible study investigators will be contacted to verify eligibility. Participant level exclusion criteria will be applied during the analysis. The main investigator and/or the corresponding author from any eligible study will be approached via email to participate in the Cribss IPD meta-analysis study. If there is no reply, other co-authors of the published manuscript will be subsequently approached.

For peer review only - http://bmjopen.bmj.com/site/about/guidelines.xhtml

For peer review only

Data acquisition and data management

The data centre is located in the Department of Obstetrics and Gynaecology at the University of Auckland, New Zealand, who will manage transferring and sharing of data. A detailed data management plan has been reviewed and agreed by all Cribss members.

Each eligible study lead investigator will be asked to provide de-identified individual level participant data for each participant enrolled in their study. Some indirect potential identifiers (eg, age, ethnicity) are essential demographic characteristics, and will be required. A study ID for each participant will be retained as this is essential for data integrity checking and data cleaning. Each study investigator will also be asked to provide metadata (such as questionnaires, data collection forms, data dictionaries) and study-level data to explain the variables, and data on the study representativeness (Table 1).

The anonymised data in a common format (eg, cvs., xls. or other formats that can be converted by the Cribss data centre) will be requested for transfer via the University of Auckland institutional Seafile file syncronisation and share platform or equivalent secure means. The Seafile platform has built-in file encryption. Files are encrypted before syncing to the server. User authentication is needed to access the files [23].

The anonymised dataset from each participating study will be checked for data integrity. This will include: 1) checking data range and outliers, 2) clarifying missing data, 3) identifying invalid values, 4) detecting duplicates, and 5) verifying internal consistency where appropriate. Reports of discrepancies will be generated and sent to each participating study investigator for further verification or correction where necessary.

After appropriate data cleaning, the individual participating study investigators will confirm and sign-off on their own dataset before it is merged into the IPD database. New variables

For peer review only - http://bmjopen.bmj.com/site/about/guidelines.xhtml

will be generated following a set of consistent harmonisation rules that will be decided by the Cribss group. An IPD data dictionary will be created to document the details of variables (including variable names, type, explanation, and validation rules) to help other users to understand the dataset.

Data items

We aim to collect the following data items from each participating study (Table 1).

Table 1 Data items will be requested from participating studies

**Study level information**
1. Study inclusion and exclusion criteria<br>2. Matching method of cases and controls<br>3. Time period of recruitment<br>4. Number of cases and controls<br>5. Informed consent procedure<br>6. Study participant representativeness (eg, minimal demographic data comparison between participant and eligible non-participant, or between participants and a relevant comparison of a maternity care population)
**Participant level information**
**A. Maternal characteristics**
1. Unique study ID<br>2. Maternal demographic details including: age, ethnicity<br>3. Past obstetric history<br>4. Maternal height<br>5. Earliest available maternal weight in the study pregnancy<br>6. Gestation at earliest available weight<br>7. Last available maternal weight in current pregnancy

For peer review only

8. Gestation at last available weight<br>9. Study centre (if the study was conducted in more than one centre)<br>10. Highest completed education level at the time of recruitment<br>11. Marital status at the time of recruitment<br>12. Pre-existing medical conditions and medical conditions during the study pregnancy<br>13. Smoking status before and during the study pregnancy<br>14. Exposure to second-hand smoke before and during the study pregnancy<br>15. Alcohol consumption before and during the study pregnancy<br>17. Recreational drug usage before and during the study pregnancy
**B. Maternal sleep practices and fetal movement data in every available time frame**
1. Going-to-sleep position<br>2. Sleep duration<br>3. Number of times getting up during the night (eg, to go to the toilet)<br>4. Frequency of daytime napping<br>5. Bed size<br>6. Number of people shared bed with<br>7. Self-reported details of snoring behaviour<br>8. Insomnia<br>9. Sleep quality as measured by validated questionnaire<br>10. Maternal perception of fetal movement<br>11. Advice received on fetal movement<br>12. Advice received on sleep position
**C. Antenatal care and pregnancy outcomes**
1. Gestation (gestation at enrolment for controls, and gestation at diagnosis of stillbirth for cases)<br>2. Baby sex<br>3. Baby birthweight

For peer review only - http://bmjopen.bmj.com/site/about/guidelines.xhtml

4. Gestation for calculating birthweight centile<br>5. Birthweight centile per original study standards<br>6. Type of facility of baby's birth<br>7. Gestation at earliest ultrasound<br>8. Blood pressure and gestation at measurement<br>9. Type of maternity provider<br>10. Number of antenatal visits in each trimester<br>11. Ultrasound scans (first trimester scan, anatomy scan and third trimester growth scan(s))<br>12. Antenatal vaginal bleeding<br>13. Hospital admission(s)<br>14. Use of antibiotics<br>15. Nutritional supplements<br>16. Clinical suspicion of fetal growth restriction (FGR) /SGA<br>17. Management of clinically suspected FGR/SGA<br>18. Laboratory tests for glucose metabolism (including polycose glucose challenge test, haemoglobin A1c and oral glucose tolerance test), hepatitis B status and blood group and the gestation that the tests were conducted.
**D. Stillbirth cases specific data**
1. Time of day mother thought the baby died<br>2. The reason that the mother thought something was wrong with the pregnancy<br>3. The reason that the mother saw a health practitioner at the diagnosis of stillbirth<br>4. Maternal decision on postmortem<br>5. Placental pathology results<br>6. The Perinatal Society of Australia and New Zealand (PSANZ) coding for classification of cause of stillbirth

Outcome measures

For peer review only

The primary outcome is late stillbirth, using the WHO recommended definition for stillbirth for international comparison: “a baby born with no signs of life at or after 28 weeks' gestation” [24]. Intrapartum stillbirth will be included in the analysis with the rationale that supine going-to-sleep position may result in a vulnerable baby that is unable to tolerate labour.

Risk of bias assessment

Risk of bias for non-randomised studies will be assessed in duplicate and independently by two investigators from the Cribss group, using Risk of Bias In Non-randomized Studies – of Exposure (ROBINS-E) assessment tool [25]. The assessment results will be compared. Any disagreement will be resolved by discussion or by a third reviewer.

Statistical analysis plan

A detailed statistical analysis plan will be prepared by the Cribss data centre group and reviewed, agreed upon and published by the Cribss group prior to the analysis. All going-to-sleep positions will be compared to left sided going-to-sleep position as the reference group. The last available going-to-sleep position during pregnancy (within two weeks before stillbirth in cases) will be harmonised and used for the primary objectives.

An individual participant data (IPD) analysis will be performed. A one stage approach to analysis will be taken so that the individual participant data from all eligible studies are included in a single model. Logistic regression models will be used for the binary outcome (late stillbirth). A fixed study effect and a study site effect will be included in the model specification as strata. Univariable analysis will be performed to evaluate the association between sleep position and late stillbirth risk. The interaction between sleep position and factors indicating a vulnerable pregnancy will be assessed in bi-variable models. A multivariable model will be developed incorporating previously reported confounders and any significant interaction terms, once it has been established what cofounders can be controlled

For peer review only - http://bmjopen.bmj.com/site/about/guidelines.xhtml

for consistently across studies. Estimate of risk will be reported as odds ratio and 95% confidence intervals.

If an important confounder is not available for one or more studies, sensitivity analysis will be conducted, with and without these studies, to compare risk estimates. If there are any controls who reported their pregnancy going-to-sleep position after they have given birth, sensitivity analysis will be conducted without these controls. Where sufficient data exist, all analysis will be also conducted in term and preterm subgroups. For missing data in each individual study, no imputation will be carried out. Statistical analyses will be performed using SAS (SAS Institute Inc., Cary NC USA).

ETHICS AND DISSEMINATION

The IPD meta-analysis has obtained central ethics approval from the New Zealand Health and Disability Ethics Committee, ref: NTX/06/05/054/AM06. The participating studies retain the right to withdraw their data from the analysis at any time.

Final IPD results will be presented to the nominated representative from each participating study prior to publication and public dissemination. Interpretation of the results will be discussed with the Cribss consumer representatives. Results of the study will be published in peer-reviewed journals and presented at national and international conferences. For the publications from the main questions, every Cribss member will participate in the manuscript preparation and editing. Authorship will be guided by the recommendations of the International Committee of Medical Journal Editors.

CONCLUSION

Cribss is the first IPD meta-analysis to evaluate the current evidence of the relationship between maternal going-to-sleep position and late stillbirth. The study will allow assessment of important interactions that cannot be tested in standard, aggregate data meta-analysis.

For peer review only

The overall goal of Cribss is to reduce late stillbirth by developing high quality data based evidence- to inform public health messages about optimal late pregnancy sleep practices. This IPD meta-analysis may identify sub-groups of women at greater risk (such as those with known SGA fetuses, who continue to smoke during pregnancy or are overweight) and thus develop evidence that can be used to tailor public health messages.

AUTHORS' CONTRIBUTION: ML, JMDT, RSC, AG, CRG, AEPH, TS, EAM, LMEM, LA conceptualised the study. ML, JMDT, RSC, AG, CRG, AEPH, TS, VC, VB, EAM, LMEM, LA have participated in study design and funding application. ML drafted the manuscript. RSC drafted appendix1. LA, JMDT, RSC, AG, CRG, AEPH, TS, EAM, LMEM, critically revised the manuscript. ML, JMDT, RSC, AG, CRG, AEPH, TS, VC, VB, EAM, LMEM, LA have read and approved submission of the final manuscript. LMEM is the guarantor of the review.

FUNDING STATEMENT: This work was supported by 2016 Trans-Tasman Research Funding Grant, by Cure Kids and Red Nose, Australia (Grant 6601). Funder has no role in developing the protocol.

COMPETING INTERESTS STATEMENT: The authors declare that they have no competing interests.

REFERENCES:

1.Lawn JE, Blencowe H, Waiswa P, Amouzou A, Mathers C, Hogan D, et al. Stillbirths: rates, risk factors, and acceleration towards 2030. Lancet. 2016 Feb 06;387(10018):587-603.

2. Flenady V, Wojcieszek AM, Middleton P, Ellwood D, Erwich JJ, Coory M, et al. Stillbirths: recall to action in high-income countries. Lancet. 2016 Feb 13;387(10019):691-702.

3. Manktelow BN SL, Seaton SE, Hyman-Taylor P, Kurinczuk JJ, Field DJ, Smith PW, Draper ES, on behalf of the MBRRACE-UK Collaboration. MBRRACE-UK Perinatal Mortality Surveillance Report, UK Perinatal Deaths for Births from January to December 2014. .

For peer review only - http://bmjopen.bmj.com/site/about/guidelines.xhtml

For peer review only

368 Leicester: The Infant Mortality and Morbidity Studies, Department of Health Sciences,
369 University of Leicester, 2016. Report No.
370 4. Stillbirth Collaborative Research Network Writing G. Association between stillbirth and risk
371 factors known at pregnancy confirmation. Jama. 2011 Dec 14;306(22):2469-79.
372 5. Smith GC. Screening and prevention of stillbirth. Best practice & research Clinical
373 obstetrics & gynaecology. 2017 Jan;38:71-82.
374 6. Stacey T, Thompson JM, Mitchell EA, Ekeroma AJ, Zuccollo JM, McCowan LM.
375 Association between maternal sleep practices and risk of late stillbirth: a case-control study.
376 Bmj. 2011;342:d3403.
377 7. Gordon A, Raynes-Greenow C, Bond D, Morris J, Rawlinson W, Jeffery H. Sleep position,
378 fetal growth restriction, and late-pregnancy stillbirth: the Sydney stillbirth study. Obstetrics
379 and gynecology. 2015 Feb;125(2):347-55.
380 8. Owusu JT, Anderson FJ, Coleman J, Oppong S, Seffah JD, Aikins A, et al. Association of
381 maternal sleep practices with pre-eclampsia, low birth weight, and stillbirth among Ghanaian
382 women. International journal of gynaecology and obstetrics: the official organ of the
383 International Federation of Gynaecology and Obstetrics. 2013 Jun;121(3):261-5.
384 9. McCowan LME, Thompson JMD, Cronin RS, Li M, Stacey T, Stone PR, et al. Going to
385 sleep in the supine position is a modifiable risk factor for late pregnancy stillbirth; Findings
386 from the New Zealand multicentre stillbirth case-control study. PloS one.
387 2017;12(6):e0179396.
388 10. Jeffreys RM, Stepanchak W, Lopez B, Hardis J, Clapp JF, 3rd. Uterine blood flow during
389 supine rest and exercise after 28 weeks of gestation. BJOG : an international journal of
390 obstetrics and gynaecology. 2006 Nov;113(11):1239-47.
391 11. Khatib N, Weiner Z, Beloosesky R, Vitner D, Thaler I. The effect of maternal supine
392 position on umbilical and cerebral blood flow indices. European journal of obstetrics,
393 gynecology, and reproductive biology. 2014 Apr;175:112-4.

For peer review only - http://bmjopen.bmj.com/site/about/guidelines.xhtml

12. Stone PR, Burgess W, McIntyre JP, Gunn AJ, Lear CA, Bennet L, et al. Effect of maternal position on fetal behavioural state and heart rate variability in healthy late gestation pregnancy. The Journal of physiology. 2017 Feb 15;595(4):1213-21.

13. Milsom I, Forssman L. Factors influencing aortocaval compression in late pregnancy. American journal of obstetrics and gynecology. 1984 Mar 15;148(6):764-71.

14. Leppanen T, Toyras J, Muraja-Murro A, Kupari S, Tiihonen P, Mervaala E, et al. Length of Individual Apnea Events Is Increased by Supine Position and Modulated by Severity of Obstructive Sleep Apnea. Sleep disorders. 2016;2016:9645347.

15. Fung AM, Wilson DL, Lappas M, Howard M, Barnes M, O'Donoghue F, et al. Effects of maternal obstructive sleep apnoea on fetal growth: a prospective cohort study. PloS one. 2013;8(7):e68057.

16. Franklin KA, Holmgren PA, Jonsson F, Poromaa N, Stenlund H, Svanborg E. Snoring, pregnancy-induced hypertension, and growth retardation of the fetus. Chest. 2000 Jan;117(1):137-41.

17. Flenady V, Koopmans L, Middleton P, Froen JF, Smith GC, Gibbons K, et al. Major risk factors for stillbirth in high-income countries: a systematic review and meta-analysis. Lancet. 2011 Apr 16;377(9774):1331-40.

18. Warland J, Mitchell EA. A triple risk model for unexplained late stillbirth. BMC pregnancy and childbirth. 2014 Apr 14;14:142.

19. Stewart LA, Clarke M, Rovers M, Riley RD, Simmonds M, Stewart G, et al. Preferred Reporting Items for Systematic Review and Meta-Analyses of individual participant data: the PRISMA-IPD Statement. Jama. 2015 Apr 28;313(16):1657-65.

20. Riley RD, Lambert PC, Abo-Zaid G. Meta-analysis of individual participant data: rationale, conduct, and reporting. Bmj. 2010 Feb 05;340:c221.

21. Debray TP, Riley RD, Rovers MM, Reitsma JB, Moons KG, Cochrane IPDM-aMg. Individual participant data (IPD) meta-analyses of diagnostic and prognostic modeling studies: guidance on their use. PLoS medicine. 2015 Oct;12(10):e1001886.

For peer review only

For peer review only - http://bmjopen.bmj.com/site/about/guidelines.xhtml

22. Ahmed I, Debray TP, Moons KG, Riley RD. Developing and validating risk prediction models in an individual participant data meta-analysis. BMC medical research methodology. 2014 Jan 08;14:3.

23. Tudur Smith C, Hopkins C, Sydes MR, Woolfall K, Clarke M, Murray G, et al. How should individual participant data (IPD) from publicly funded clinical trials be shared? BMC medicine. 2015 Dec 17;13:298.

24. WHO. Maternal, newborn, child and adolescent health: Data, statistics and epidemiology: WHO; [cited 2016 21 November 2016]. Available from: http://www.who.int/maternal_child_adolescent/epidemiology/stillbirth/en/.

25. Morgan R. The ROBINS-E tool (Risk Of Bias In Non-randomized Studies - of Exposures): University of Bristol; 2017 [cited 2017 20th October]. Available from: http://www.bristol.ac.uk/population-health-sciences/centres/cresyda/barr/riskofbias/robins-e/.

For peer review only

Appendix 1:

Search strategy for the Collaborative IPD of Sleep and Stillbirth (Cribss) study

Databases or search engines that will be used

A search of the databases: MEDLINE, EMBASE, LILACS, Web of Science, OpenGrey, and Google Scholar, will be conducted, for the purpose of locating published research about an association between maternal sleep position and late pregnancy stillbirth. We will also access WHO International Clinical Trials Registry Platform to identify any ongoing and registered trials. Proceedings from International Stillbirth Alliance (ISA) annual conferences and The International Society for the Study and Prevention of Perinatal and Infant Death (ISPID) international conferences will be manually searched. Published perinatal conference abstracts will be identified through the above database searches. Experts in the field and the collaborative group will be asked for their knowledge of any unpublished studies.

Limits applied

To increase the likelihood of identifying all relevant studies, the reference lists of all retrieved articles will be hand searched. No language restriction will be applied.

List the search terms used

Three search terms will be used to search the databases with the article title, abstracts and body all searched. The search terms are:

- stillbirth
- fetal death
- sleep

and synonyms. The search terms will be tested to check that they effectively located the types of articles that are consistent with the inclusion criteria prior to conducting the search in all engines.

Document the search process

The following search was conducted sequentially using the search terms in MEDLINE on 20th November 2016.

Search engine		Search terms	# Retrieved:
**MEDLINE**			
MEDLINE	1	Stillbirth/	3851
MEDLINE	2	(stillbirth* or still-birth* or stillborn* or still-born*).ti,ab,kf.	13691
MEDLINE	3	Fetal Death/	24585
MEDLINE	4	((fetal or foetal or fetus or foetus) adj death*).ti,ab,kf.	8769
MEDLINE	5	((fetal or foetal or fetus or foetus) adj3 (loss or losses)).ti,ab,kf.	4804
MEDLINE	6	Perinatal Death/	860
MEDLINE	7	((perinatal or peri-natal) adj death*).ti,ab,kf.	4007
MEDLINE	8	((prenatal or pre-natal or intrauterine or intra-uterine or antepartum or ante-partum or antenatal or ante-natal) adj death*).ti,ab,kf.	2026
MEDLINE	9	or/1-8	46353
MEDLINE	10	Sleep/	46957
MEDLINE	11	((sleep or sleeping) adj (position* or practice* or posture*)).ti,ab,kf.	1354
MEDLINE	12	maternal sleep*.ti,ab,kf.	139
MEDLINE	13	or/10-12	47711
MEDLINE	14	9 and 13	23

For peer review only

PRISMA-P (Preferred Reporting Items for Systematic review and Meta-Analysis Protocols) 2015 checklist: recommended items to address in a systematic review protocol*

Section and topic	Item No	Checklist item	Check
**ADMINISTRATIVE INFORMATION**			
Title:			
Identification	1a	Identify the report as a protocol of a systematic review	Yes, P1, line 2
Update	1b	If the protocol is for an update of a previous systematic review, identify as such	na
Registration	2	If registered, provide the name of the registry (such as PROSPERO) and registration number	Yes, P3, line 60
Authors:			
Contact	3a	Provide name, institutional affiliation, e-mail address of all protocol authors; provide physical mailing address of corresponding author	Yes, P2, line 4-36
Contributions	3b	Describe contributions of protocol authors and identify the guarantor of the review	Yes, P17, line 346-350
Amendments	4	If the protocol represents an amendment of a previously completed or published protocol, identify as such and list changes; otherwise, state plan for documenting important protocol amendments	na
Support:			
Sources	5a	Indicate sources of financial or other support for the review	Yes, P17, line 353
Sponsor	5b	Provide name for the review funder and/or sponsor	Yes, P17, line 353
Role of sponsor or funder	5c	Describe roles of funder(s), sponsor(s), and/or institution(s), if any, in developing the protocol	Yes, P17, line 353
**INTRODUCTION**			
Rationale	6	Describe the rationale for the review in the context of what is already known	Yes, P5, line 80-166
Objectives	7	Provide an explicit statement of the question(s) the review will address with reference to participants, interventions, comparators, and outcomes (PICO)	Yes, P8, line 163
**METHODS**			
Eligibility criteria	8	Specify the study characteristics (such as PICO, study design, setting, time frame) and report characteristics (such as years considered, language, publication status) to be used as criteria for eligibility for the review	Yes, P9, line 195
Information sources	9	Describe all intended information sources (such as electronic databases, contact with study authors, trial registers or other grey literature sources) with planned dates of coverage	Yes, P9-10, line 214

For peer review only

Search strategy	10	Present draft of search strategy to be used for at least one electronic database, including planned limits, such that it could be repeated	Yes, P9-10, line 214 and appendix 1
Study records:			
Data management	11a	Describe the mechanism(s) that will be used to manage records and data throughout the review	Yes, P11, line 247-277
Selection process	11b	State the process that will be used for selecting studies (such as two independent reviewers) through each phase of the review (that is, screening, eligibility and inclusion in meta-analysis)	Yes, P10, line 236
Data collection process	11c	Describe planned method of extracting data from reports (such as piloting forms, done independently, in duplicate), any processes for obtaining and confirming data from investigators	Yes, P11, line 247-277
Data items	12	List and define all variables for which data will be sought (such as PICO items, funding sources), any pre-planned data assumptions and simplifications	Yes, line 279-283 (table 1)
Outcomes and prioritization	13	List and define all outcomes for which data will be sought, including prioritization of main and additional outcomes, with rationale	Yes, P14-15, line 284
Risk of bias in individual studies	14	Describe anticipated methods for assessing risk of bias of individual studies, including whether this will be done at the outcome or study level, or both; state how this information will be used in data synthesis	Yes, P15, line 290
Data synthesis	15a	Describe criteria under which study data will be quantitatively synthesised	Yes, P15, line 296
	15b	If data are appropriate for quantitative synthesis, describe planned summary measures, methods of handling data and methods of combining data from studies, including any planned exploration of consistency (such as I^2, Kendall's τ)	Yes, P15, line 296
	15c	Describe any proposed additional analyses (such as sensitivity or subgroup analyses, meta-regression)	Yes, P16, line 315
	15d	If quantitative synthesis is not appropriate, describe the type of summary planned	na
Meta-bias(es)	16	Specify any planned assessment of meta-bias(es) (such as publication bias across studies, selective reporting within studies)	Yes, P16, line 315
Confidence in cumulative evidence	17	Describe how the strength of the body of evidence will be assessed (such as GRADE)	na

*** It is strongly recommended that this checklist be read in conjunction with the PRISMA-P Explanation and Elaboration (cite when available) for important clarification on the items. Amendments to a review protocol should be tracked and dated.** **The copyright for PRISMA-P (including checklist) is held by the PRISMA-P Group and is distributed under a Creative Commons Attribution Licence 4.0.**

From: Shamseer L, Moher D, Clarke M, Ghersi D, Liberati A, Petticrew M, Shekelle P, Stewart L, PRISMA-P Group. Preferred reporting items for systematic review and meta-analysis protocols (PRISMA-P) 2015: elaboration and explanation. BMJ. 2015 Jan 2;349(jan02 1):g7647.

For peer review only

For peer review only - http://bmjopen.bmj.com/site/about/guidelines.xhtml

The Collaborative IPD of Sleep and Stillbirth (Cribss): Is maternal going-to-sleep position a risk factor for late stillbirth and does maternal sleep position interact with fetal vulnerability? – An Individual Participant Data Meta-Analysis study protocol

Journal:	*BMJ Open*
Manuscript ID	bmjopen-2017-020323.R1
Article Type:	Protocol
Date Submitted by the Author:	03-Feb-2018

Complete List of Authors:	Li, Minglan; University of Auckland, Department of Obstetrics and Gynaecology<br>Thompron, John; University of Auckland, Paediatrics: Child and Youth Health<br>Cronin, Robin; University of Auckland, Department of Obstetrics and Gynaecology<br>Gordon, Adrienne; University of Sydney - Camden Campus, Department of Paediatrics<br>Raynes-Greenow, Camille; The University of Sydney, School of Public Health<br>Heazell, Alexander; University of Manchester, Maternal and Fetal Health Research Centre<br>Stacey, Tomasina; University of Leeds, School of Healthcare<br>Culling, Vicki ; Vicki Culling Associates<br>Bowring, Victoria; Stillbirth Foundation Australia<br>Mitchell, Edwin; University of Auckland, Paediatrics<br>McCowan, Lesley; University of Auckland<br>Askie, Lisa; University of Sydney, National Health and Medical Research Council Clinical Trials Centre

**Primary Subject Heading**:	Obstetrics and gynaecology
Secondary Subject Heading:	Epidemiology
Keywords:	Stillbirth, Sleep position, Individual participant data meta-analysis, Small for gestational age, Fetal movement

SCHOLARONE™ Manuscripts

For peer review only - http://bmjopen.bmj.com/site/about/guidelines.xhtml

The Collaborative IPD of Sleep and Stillbirth (Cribss): Is maternal going-to-sleep position a risk factor for late stillbirth and does maternal sleep position interact with fetal vulnerability? – An Individual Participant Data Meta-Analysis study protocol

Minglan Li[1] m.li@auckland.ac.nz

John MD Thompson[1,2] j.thompson@auckland.ac.nz

Robin S Cronin[1] r.cronin@auckland.ac.nz

Adrienne Gordon[3,4] adrienne.gordon@sydney.edu.au

Camille Raynes-Greenow[5] camille.raynes-greenow@sydney.edu.au

Alexander E P Heazell[6,7] alexander.heazell@manchester.ac.uk

Tomasina Stacey[8] t.stacey@leeds.ac.uk

Vicki Culling[9] vicki@vca.co.nz

Victoria Bowring[10] victoria@stillbirthfoundation.org.au

Edwin A Mitchell[2] e.mitchell@auckland.ac.nz

Lesley ME McCowan[1] l.mccowan@auckland.ac.nz

Lisa Askie[11] lisa.askie@ctc.usyd.edu.au

1. Department of Obstetrics and Gynaecology, University of Auckland, Auckland, New Zealand. 2. Department of Paediatrics and Child Health, University of Auckland, Auckland, New Zealand. 3. Department of Newborn Care, Royal Prince Alfred Hospital Women and Babies, Sydney, Australia. 4. The University of Sydney, Charles Perkins Centre, University of Sydney, Sydney, NSW, Australia. 5. The University of Sydney, Sydney School of Public Health, Sydney, NSW, Australia. 6. Maternal and Fetal Health Research Centre, Division of Developmental Biomedicine, Faculty of Medical and Human Sciences, University of Manchester, UK. 7. St. Mary's Hospital, Central Manchester University Hospitals NHS Foundation Trust, Manchester Academic Health Science Centre, Manchester, UK. 8. School of Healthcare, University of Leeds, Leeds, UK. 9. Vicki Culling Associates, Auckland, New

For peer review only - http://bmjopen.bmj.com/site/about/guidelines.xhtml

Zealand. 10. Stillbirth Foundation, Australia. 11. National Health and Medical Research Council Clinical Trials Centre, University of Sydney, Camperdown, Australia.

Co-responding author:

Minglan Li

Department of Obstetrics and Gynaecology,

University of Auckland,

Private Bag 92019

Auckland, New Zealand

For peer review only

For peer review only - http://bmjopen.bmj.com/site/about/guidelines.xhtml

ABSTRACT

Introduction: Accumulating evidence has shown an association between maternal supine going-to-sleep position and stillbirth in late pregnancy. Advising women not to go to sleep on their back can potentially reduce late stillbirth rate by 9%. However, the association between maternal right-sided going-to-sleep position and stillbirth is inconsistent across studies. Furthermore, individual studies are underpowered to investigate interactions between maternal going-to-sleep position and fetal vulnerability, which is potentially important for producing clear and tailored public health messages on safe going-to-sleep position. We will use individual participant data (IPD) from existing studies to assess whether right-side and supine going-to-sleep positions are independent risk factors for late stillbirth and test the interaction between going-to-sleep position and fetal vulnerability.

Methods and Analysis: An IPD meta-analysis approach will be utilised using the Cochrane Collaboration-endorsed methodology. We will identify case-control and prospective cohort studies and randomised trials which collected maternal going-to-sleep position data and pregnancy outcome data that included stillbirth. The primary outcome is stillbirth. A one stage procedure meta-analysis, stratified by study with adjustment of a priori confounders will be carried out.

Ethics and dissemination: The IPD meta-analysis has obtained central ethics approval from the New Zealand Health and Disability Ethics Committee, ref: NTX/06/05/054/AM06. Individual studies should also have ethical approval from relevant local ethics committees. Interpretation of the results will be discussed with consumer representatives. Results of the study will be published in peer-reviewed journals and presented at international conferences.

Systematic review registration: PROSPERO registration number: CRD42017047703

STRENGTHS AND LIMITATIONS OF THIS STUDY

- Late stillbirth is a rare event in high-income countries, and individual participant data meta-analysis of several studies can yield a sufficiently large sample size for exploring interactions and subgroup analysis that are difficult to undertake within a single study.

For peer review only - http://bmjopen.bmj.com/site/about/guidelines.xhtml

- There is no restriction on language or countries where the study was conducted, therefore the results from this study are likely to be generalisable.
- Service users will oversee the conduct of the study. Their involvement will help to design appropriate research questions and will help the implementation and translation of the research outcomes.
- One limitation of the study is that the maternal going-to-sleep positions are likely to be self-reported.

For peer review only

For peer review only - http://bmjopen.bmj.com/site/about/guidelines.xhtml

INTRODUCTION

Stillbirth, the death of a baby before birth, is a major global burden affecting more than 2.6 million families per year [1]. In high-income countries, the rate of late stillbirth (28 weeks or greater) varies widely from 1.3 to 8.8 per 1,000 births [2] and is approximately twice as common as neonatal death [3]. Importantly, the annual rate of reduction for neonatal death is twice that of stillbirth [2]. The variations between countries suggest it is possible to further reduce late stillbirth. Importantly, maternal characteristics present in early pregnancy only explain a small amount of the risk for late stillbirth [4]. Therefore, significant reductions in late stillbirth require identification of additional maternal risk factors amenable to modification during pregnancy [5].

Accumulating evidence suggests that supine going-to-sleep position may be a modifiable risk factor for stillbirth in late pregnancy. Stacey *et al.* first reported an association between going-to-sleep position and late stillbirth, with women who did not go-to-sleep on their left side, the night before the baby was suspected to have died, having an increased odds of stillbirth [6]. Among non-left sided sleepers, the odds were greater in women who went to sleep supine; and there was also a borderline increase in odds in women who went to sleep on their right side [6]. Similar associations between supine going-to-sleep position and late stillbirth have since been reported by several studies [7-9]. In addition to the epidemiologic evidence, a number of physiological studies have suggested that the relationship between supine going-to-sleep position and late stillbirth is biologically plausible. Significant hemodynamic changes in maternal and fetal circulation have been observed in relation to maternal position in late pregnancy, with decreased maternal cardiac output and uterine blood flow [10], and pulsatility index in the fetal middle cerebral artery (a surrogate for fetal hypoxia) [11] seen in maternal supine position when compared to left position. A recent study by Stone *et al*. has shown that when the mother is in the supine position, the fetus spends more time in behavioural state 1 (fetal quiescence) and less time in active fetal behavioural state 4, compared to when the mother is on her left side, indicating supine

For peer review only - http://bmjopen.bmj.com/site/about/guidelines.xhtml

position may be a mild hypoxic stressor [12]. It was hypothesised that these physiological changes associated with supine position are related to the direct compression of the inferior vena cava by the gravid uterus [13]. Furthermore, supine sleep position is also associated with sleep disturbed breathing and obstructive sleep apnoea [14], which have also been associated with pregnancy complications such as pre-eclampsia, fetal growth restriction [15], and gestational diabetes [15, 16]. These pregnancy complications are known risk factors for stillbirth [17], and might represent another mechanism that contributes to the association between supine going-to-sleep position and late stillbirth.

The findings from the epidemiological studies combined with the supportive physiological evidence suggest that the association between supine sleep position and late stillbirth is likely to be causal. Informing pregnant women and their healthcare providers about optimal going-to-sleep position in late pregnancy is a strategy that may reduce stillbirth and is potentially harmless. Therefore, there is an urgent need to assess the accumulated evidence to develop a public health campaign. However, there are some unanswered questions that are critical for developing clear public health messages. Firstly, it is unclear whether right sided going-to-sleep position is a risk factor for late stillbirth. A borderline increase in risk was reported with right side compared to left side going-to-sleep position in the Stacey *et al.* study. However, this association was not found in other studies [7, 9]. The inconsistent finding of right side going-to-sleep position warrants further clarification so that clear advice about whether women should be advised to go-to-sleep on either side or only on their left side can be developed. Secondly, there is no evidence whether there are groups of women who are at elevated risk when they go-to-sleep in a suboptimal position (such as those who smoke, are overweight or have small babies etc.) and how other stillbirth risk factors interact with sleep position. Stillbirth is the end point of diverse pathological processes. Multiple risk factors and pathological events can contribute at different time points and cumulatively lead to the final event. Our research group has hypothesised a triple-risk framework for late stillbirth that cannot be explained by one risk factor or condition alone [18]. We speculate

For peer review only - http://bmjopen.bmj.com/site/about/guidelines.xhtml

that three groups of factors namely maternal factors (eg, obesity, smoking), fetal and placental factors (eg, a small for gestational age (SGA) fetus) and an additional stressor(s) (eg, reduced uterine blood flow associated with supine position) in themselves may be insufficient to cause the death, but their combination may have a lethal effect [18]. Individual stillbirth case control studies published to date have insufficient power to explore fully the interactions between supine going-to-sleep position, markers of fetal vulnerability and adverse maternal factors. Furthermore, it is important to explore other factors that may also be associated with supine sleep position such as SGA, reduced fetal movements and sleep disturbed breathing, as this may provide insights into the potential mechanism of risk associated with the supine position.

The Collaborative IPD Sleep and Stillbirth (Cribss) group was established in December 2016. We aim to synthesise the current evidence about going-to-sleep position and stillbirth risk. Additionally we will address the above unanswered questions by combining and analysing the individual participant data from all available studies in an individual participant data (IPD) meta-analysis. IPD meta-analysis is considered the gold standard approach to evidence synthesis as it has the potential to improve the precision and reliability of the results obtained from individual studies [19]. In contrast to the traditional approach of meta-analysis, which extracts summary (aggregate) data from study publications, an IPD meta-analysis uses line-by-line original data sourced directly from the researchers responsible for the relevant studies. An IPD meta-analysis involves the central collection, checking, harmonisation and re-analysis of the original data of all eligible participants from each of the available studies. With proper quality assessment and standardisation processes, an IPD meta-analysis can model complex relationships, which traditional meta-analyses are not able to do [20]. It is particularly useful in evaluating multi-factorial frameworks by evaluating critical outcome determinants and their interactions.

For peer review only - http://bmjopen.bmj.com/site/about/guidelines.xhtml

OBJECTIVES

The main questions to be addressed by the Cribss IPD meta-analysis are:

1. Is maternal going-to-sleep position associated with late stillbirth?
2. Are indicators of fetal vulnerability, including: maternal obesity, SGA, maternal smoking, maternal second-hand tobacco exposure, substance use, alcohol consumption, maternal medical conditions (including pre-existing hypertension and diabetes), and maternal perception of fetal movements associated with late stillbirth, and does maternal going-to-sleep position interact with indicators of fetal vulnerability to influence the risk of late stillbirth? Does birthweight centile interact with maternal going-to-sleep position to influence the risk of late stillbirth?

Secondary questions to be addressed by the first cycle of Cribss IPD meta-analysis are:

1. Is sleep disturbed breathing associated with late stillbirth? Is (are) going-to-sleep position(s) associated with greater risk of late stillbirth in women with sleep disturbed breathing? Is sleep disturbed breathing a moderator for sleep position in relation to late stillbirth?
2. Are factors that may influence vena caval compression (eg, long sleep duration, sleeping during the day, restless legs,) associated with risk of late stillbirth? Do these factors interact with going-to-sleep position?
3. Do women who report they received advice about sleep position have lower risk of late stillbirth compared with women who did not receive such advice?
4. Do women who report they received advice about awareness of fetal movements have a lower risk of late stillbirth than women who did not receive such advice?

METHODS AND ANALYSIS

This study will apply an IPD meta-analysis approach, and will follow the methodology endorsed by the Cochrane Collaboration where applicable [21, 22]. We will adhere to the Preferred Reporting Items for Systematic Reviews and Meta-analysis (PRISMA) IPD statement for reporting findings. The study will be conducted by the Collaborative IPD Sleep

For peer review only - http://bmjopen.bmj.com/site/about/guidelines.xhtml

and Stillbirth (Cribss) group which comprises the participating study investigators, an IPD expert, and consumer representatives. The coordination centre is located in the department of Obstetrics and Gynaecology at the University of Auckland, Auckland, New Zealand. We have registered the IPD Study with the PROSPERO international prospective register of systematic reviews (CRD42017047703).

Eligibility criteria

Study inclusion criteria (regardless of whether the study is published or unpublished):

1. Case-control and prospective cohort studies which collected:
    - Maternal going-to-sleep position during pregnancy and
    - Pregnancy outcome that included stillbirth and
    - Aimed to recruit controls with an on-going pregnancy at similar gestation to the cases
2. Randomised controlled trials which collected:
    - Maternal going-to-sleep position during pregnancy and
    - Pregnancy outcome data that included stillbirth

Participant level exclusion criteria:

- Multiple pregnancy in the third trimester
- Major congenital abnormality at study entry or major congenital abnormality as a cause of death found post study entry or post-randomisation in randomised controlled trials
- Gestation less than 28 weeks when last sleep position data during pregnancy was collected
- Termination of pregnancy at greater than or equal to 28 weeks
- Received study intervention that might have an impact on going-to-sleep position.

Information sources and search strategy

For peer review only

We will develop the search strategy according to the Cochrane Collaboration guidelines prior to the initial literature search. A search of the databases: MEDLINE, EMBASE, LILACS, Web of Science, OpenGrey, and Google Scholar will be conducted for the purpose of locating published research about an association between maternal sleep position and late pregnancy stillbirth. We will also access the WHO International Clinical Trials Registry Platform to identify any ongoing and registered trials. Proceedings from International Stillbirth Alliance (ISA) annual conferences and The International Society for the Study and Prevention of Perinatal and Infant Death (ISPID) international conferences will be manually searched. Published perinatal conference abstracts will also be identified through the above database searches. Experts in the field and the collaborative group will be asked about their knowledge of any unpublished studies. To increase the likelihood of identifying all relevant studies the reference lists of all retrieved articles will be hand searched. No language restriction will be applied.

Four search terms will be used to search the databases with the article title, abstracts and body all searched. The search terms are: 'stillbirth', 'fetal death', 'perinatal death' and 'sleep' and synonyms. The search terms will be tested to check that they effectively located the types of articles that are consistent with the inclusion criteria prior to conducting the search in all engines. An example of a detailed MEDLINE search strategy is presented in supplementary appendix 1.

Selection process

Study eligibility will be assessed independently by two members of the Cribss group, any disagreements will be adjudicated by a third member. Eligibility assessment will be based on published protocols, method sections from publications, and unpublished protocols and, or study information requested from potential eligible study investigators. All potential eligible study investigators will be contacted to verify eligibility. Participant level exclusion criteria will be applied during the analysis. The main investigator and/or the corresponding author from

For peer review only - http://bmjopen.bmj.com/site/about/guidelines.xhtml

any eligible study will be approached via email to participate in the Cribss IPD meta-analysis study. If there is no reply, other co-authors of the published manuscript will be subsequently approached.

Data acquisition and data management

The data centre is located in the Department of Obstetrics and Gynaecology at the University of Auckland, New Zealand, who will manage transferring and sharing of data. A detailed data management plan has been reviewed and agreed by all Cribss members.

Each eligible study lead investigator will be asked to provide de-identified individual level participant data for each participant enrolled in their study. Some indirect potential identifiers (eg, age, ethnicity) are essential demographic characteristics, and will be required. A study ID for each participant will be retained as this is essential for data integrity checking and data cleaning. Each study investigator will also be asked to provide metadata (such as questionnaires, data collection forms, data dictionaries) and study-level data to explain the variables, and data on the study representativeness (Table 1).

The anonymised data in a common format (eg, cvs., xls. or other formats that can be converted by the Cribss data centre) will be requested for transfer via the University of Auckland institutional Seafile file syncronisation and share platform or equivalent secure means. The Seafile platform has built-in file encryption. Files are encrypted before syncing to the server. User authentication is needed to access the files [23].

The anonymised dataset from each participating study will be checked for data integrity. This will include: 1) checking data range and outliers, 2) clarifying missing data, 3) identifying invalid values, 4) detecting duplicates, and 5) verifying internal consistency where appropriate. Reports of discrepancies will be generated and sent to each participating study investigator for further verification or correction where necessary.

For peer review only - http://bmjopen.bmj.com/site/about/guidelines.xhtml

After appropriate data cleaning, the individual participating study investigators will confirm and sign-off on their own dataset before it is merged into the IPD database. New variables will be generated following a set of consistent harmonisation rules that will be decided by the Cribss group. An IPD data dictionary will be created to document the details of variables (including variable names, type, explanation, and validation rules) to help other users to understand the dataset.

Data items

We aim to collect the following data items from each participating study (Table 1).

Table 1 Data items will be requested from participating studies

**Study level information**
1. Study inclusion and exclusion criteria<br>2. Matching method of cases and controls<br>3. Time period of recruitment<br>4. Number of cases and controls<br>5. Informed consent procedure<br>6. Study participant representativeness (eg, minimal demographic data comparison between participant and eligible non-participant, or between participants and a relevant comparison of a maternity care population)
**Participant level information**
**A. Maternal characteristics**
1. Unique study ID<br>2. Maternal demographic details including: age, ethnicity<br>3. Past obstetric history<br>4. Maternal height

For peer review only - http://bmjopen.bmj.com/site/about/guidelines.xhtml

5. Earliest available maternal weight in the study pregnancy<br>6. Gestation at earliest available weight<br>7. Last available maternal weight in current pregnancy<br>8. Gestation at last available weight<br>9. Study centre (if the study was conducted in more than one centre)<br>10. Highest completed education level at the time of recruitment<br>11. Marital status at the time of recruitment<br>12. Pre-existing medical conditions and medical conditions during the study pregnancy<br>13. Smoking status before and during the study pregnancy<br>14. Exposure to second-hand smoke before and during the study pregnancy<br>15. Alcohol consumption before and during the study pregnancy<br>17. Recreational drug usage before and during the study pregnancy
**B. Maternal sleep practices and fetal movement data in every available time frame**
1. Going-to-sleep position<br>2. Sleep duration<br>3. Number of times getting up during the night (eg, to go to the toilet)<br>4. Frequency of daytime napping<br>5. Bed size<br>6. Number of people shared bed with<br>7. Self-reported details of snoring behaviour<br>8. Insomnia<br>9. Sleep quality as measured by validated questionnaire<br>10. Maternal perception of fetal movement<br>11. Advice received on fetal movement<br>12. Advice received on sleep position
**C. Antenatal care and pregnancy outcomes**
1. Gestation (gestation at enrolment for controls, and gestation at diagnosis of stillbirth for

For peer review only - http://bmjopen.bmj.com/site/about/guidelines.xhtml

cases)<br>2. Baby sex<br>3. Baby birthweight<br>4. Gestation for calculating birthweight centile<br>5. Birthweight centile per original study standards<br>6. Type of facility of baby's birth<br>7. Gestation at earliest ultrasound<br>8. Blood pressure and gestation at measurement<br>9. Type of maternity provider<br>10. Number of antenatal visits in each trimester<br>11. Ultrasound scans (first trimester scan, anatomy scan and third trimester growth scan(s))<br>12. Antenatal vaginal bleeding<br>13. Hospital admission(s)<br>14. Use of antibiotics<br>15. Nutritional supplements<br>16. Clinical suspicion of fetal growth restriction (FGR) /SGA<br>17. Management of clinically suspected FGR/SGA<br>18. Laboratory tests for glucose metabolism (including polycose glucose challenge test, haemoglobin A1c and oral glucose tolerance test), hepatitis B status and blood group and the gestation that the tests were conducted.
**D. Stillbirth cases specific data**
1. Time of day mother thought the baby died<br>2. The reason that the mother thought something was wrong with the pregnancy<br>3. The reason that the mother saw a health practitioner at the diagnosis of stillbirth<br>4. Maternal decision on postmortem<br>5. Placental pathology results<br>6. The Perinatal Society of Australia and New Zealand (PSANZ) coding for classification of

For peer review only - http://bmjopen.bmj.com/site/about/guidelines.xhtml

cause of stillbirth

Outcome measures

The primary outcome is late stillbirth, using the WHO recommended definition for stillbirth for international comparison: “a baby born with no signs of life at or after 28 weeks' gestation” [24]. Intrapartum stillbirth will be included in the analysis with the rationale that supine going-to-sleep position may result in a vulnerable baby that is unable to tolerate labour.

Risk of bias assessment

Risk of bias for non-randomised studies will be assessed in duplicate and independently by two investigators from the Cribss group, using Risk of Bias In Non-randomized Studies – of Exposure (ROBINS-E) assessment tool [25]. The assessment results will be compared. Any disagreement will be resolved by discussion or by a third reviewer.

Statistical analysis plan

A detailed statistical analysis plan for the main questions has been prepared by the Cribss data centre group and reviewed, and agreed upon by the Cribss group prior to the analysis (appendix 2). All going-to-sleep positions will be compared to left sided going-to-sleep position as the reference group. The last available going-to-sleep position during pregnancy (within two weeks before stillbirth in cases) will be harmonised and used for the primary objectives.

An individual participant data (IPD) analysis will be performed. A one stage approach to analysis will be taken so that the individual participant data from all eligible studies are included in a single model. Logistic regression models will be used for the binary outcome (late stillbirth). A fixed study effect and a study site effect will be included in the model specification as strata. Univariable analysis will be performed to evaluate the association

For peer review only - http://bmjopen.bmj.com/site/about/guidelines.xhtml

between sleep position and late stillbirth risk. The interaction between sleep position and factors indicating a vulnerable pregnancy will be assessed in bi-variable models. A multivariable model will be developed incorporating previously reported confounders and any significant interaction terms, once it has been established what cofounders can be controlled for consistently across studies. Estimate of risk will be reported as odds ratio and 95% confidence intervals. We will also explore if sleep apnoea is a moderator for sleep position in relation to late stillbirth using moderator analyses.

If an important confounder is not available for one or more studies, sensitivity analysis will be conducted, with and without these studies, to compare risk estimates. If there are any controls who reported their pregnancy going-to-sleep position after they have given birth, sensitivity analysis will be conducted without these controls. Where sufficient data exist, all analysis will be also conducted in term and preterm subgroups. For missing data in each individual study, no imputation will be carried out. Statistical analyses will be performed using SAS (SAS Institute Inc., Cary NC USA).

ETHICS AND DISSEMINATION

The IPD meta-analysis has obtained central ethics approval from the New Zealand Health and Disability Ethics Committee, ref: NTX/06/05/054/AM06. The participating studies retain the right to withdraw their data from the analysis at any time.

Final IPD results will be presented to the nominated representative from each participating study prior to publication and public dissemination. Interpretation of the results will be discussed with the Cribss consumer representatives. Results of the study will be published in peer-reviewed journals and presented at national and international conferences. For the publications from the main questions, every Cribss member will participate in the manuscript preparation and editing. Authorship will be guided by the recommendations of the International Committee of Medical Journal Editors.

For peer review only - http://bmjopen.bmj.com/site/about/guidelines.xhtml

CONCLUSION

Cribss is the first IPD meta-analysis to evaluate the current evidence of the relationship between maternal going-to-sleep position and late stillbirth. The study will allow assessment of important interactions that cannot be tested in standard, aggregate data meta-analysis. The overall goal of Cribss is to reduce late stillbirth by developing high quality data based evidence- to inform public health messages about optimal late pregnancy sleep practices. This IPD meta-analysis may identify sub-groups of women at greater risk (such as those with known SGA fetuses, who continue to smoke during pregnancy or are overweight) and thus develop evidence that can be used to tailor public health messages.

AUTHORS' CONTRIBUTION: ML, JMDT, RSC, AG, CRG, AEPH, TS, EAM, LMEM, LA conceptualised the study. ML, JMDT, RSC, AG, CRG, AEPH, TS, VC, VB, EAM, LMEM, LA have participated in study design and funding application. ML drafted the manuscript and appendix 2. RSC drafted appendix1. LA, JMDT, RSC, AG, CRG, AEPH, TS, EAM, LMEM, critically revised the manuscript. ML, JMDT, RSC, AG, CRG, AEPH, TS, VC, VB, EAM, LMEM, LA have read and approved submission of the final manuscript. LMEM is the guarantor of the review.

FUNDING STATEMENT: This work was supported by 2016 Trans-Tasman Research Funding Grant, by Cure Kids and Red Nose, Australia (Grant 6601). Funder has no role in developing the protocol.

COMPETING INTERESTS STATEMENT: The authors declare that they have no competing interests.

REFERENCES:

For peer review only

For peer review only - http://bmjopen.bmj.com/site/about/guidelines.xhtml

1.Lawn JE, Blencowe H, Waiswa P, Amouzou A, Mathers C, Hogan D, et al. Stillbirths: rates, risk factors, and acceleration towards 2030. Lancet. 2016 Feb 06;387(10018):587-603.

2. Flenady V, Wojcieszek AM, Middleton P, Ellwood D, Erwich JJ, Coory M, et al. Stillbirths: recall to action in high-income countries. Lancet. 2016 Feb 13;387(10019):691-702.

3. Manktelow BN SL, Seaton SE, Hyman-Taylor P, Kurinczuk JJ, Field DJ, Smith PW, Draper ES, on behalf of the MBRRACE-UK Collaboration. MBRRACE-UK Perinatal Mortality Surveillance Report, UK Perinatal Deaths for Births from January to December 2014. . Leicester: The Infant Mortality and Morbidity Studies, Department of Health Sciences, University of Leicester, 2016. Report No.

4. Stillbirth Collaborative Research Network Writing G. Association between stillbirth and risk factors known at pregnancy confirmation. Jama. 2011 Dec 14;306(22):2469-79.

5. Smith GC. Screening and prevention of stillbirth. Best practice & research Clinical obstetrics & gynaecology. 2017 Jan;38:71-82.

6. Stacey T, Thompson JM, Mitchell EA, Ekeroma AJ, Zuccollo JM, McCowan LM. Association between maternal sleep practices and risk of late stillbirth: a case-control study. Bmj. 2011;342:d3403.

7. Gordon A, Raynes-Greenow C, Bond D, Morris J, Rawlinson W, Jeffery H. Sleep position, fetal growth restriction, and late-pregnancy stillbirth: the Sydney stillbirth study. Obstetrics and gynecology. 2015 Feb;125(2):347-55.

8. Owusu JT, Anderson FJ, Coleman J, Oppong S, Seffah JD, Aikins A, et al. Association of maternal sleep practices with pre-eclampsia, low birth weight, and stillbirth among Ghanaian women. International journal of gynaecology and obstetrics: the official organ of the International Federation of Gynaecology and Obstetrics. 2013 Jun;121(3):261-5.

9. McCowan LME, Thompson JMD, Cronin RS, Li M, Stacey T, Stone PR, et al. Going to sleep in the supine position is a modifiable risk factor for late pregnancy stillbirth; Findings from the New Zealand multicentre stillbirth case-control study. PloS one. 2017;12(6):e0179396.

For peer review only

For peer review only - http://bmjopen.bmj.com/site/about/guidelines.xhtml

10. Jeffreys RM, Stepanchak W, Lopez B, Hardis J, Clapp JF, 3rd. Uterine blood flow during supine rest and exercise after 28 weeks of gestation. BJOG : an international journal of obstetrics and gynaecology. 2006 Nov;113(11):1239-47.

11. Khatib N, Weiner Z, Beloosesky R, Vitner D, Thaler I. The effect of maternal supine position on umbilical and cerebral blood flow indices. European journal of obstetrics, gynecology, and reproductive biology. 2014 Apr;175:112-4.

12. Stone PR, Burgess W, McIntyre JP, Gunn AJ, Lear CA, Bennet L, et al. Effect of maternal position on fetal behavioural state and heart rate variability in healthy late gestation pregnancy. The Journal of physiology. 2017 Feb 15;595(4):1213-21.

13. Milsom I, Forssman L. Factors influencing aortocaval compression in late pregnancy. American journal of obstetrics and gynecology. 1984 Mar 15;148(6):764-71.

14. Leppanen T, Toyras J, Muraja-Murro A, Kupari S, Tiihonen P, Mervaala E, et al. Length of Individual Apnea Events Is Increased by Supine Position and Modulated by Severity of Obstructive Sleep Apnea. Sleep disorders. 2016;2016:9645347.

15. Fung AM, Wilson DL, Lappas M, Howard M, Barnes M, O'Donoghue F, et al. Effects of maternal obstructive sleep apnoea on fetal growth: a prospective cohort study. PloS one. 2013;8(7):e68057.

16. Franklin KA, Holmgren PA, Jonsson F, Poromaa N, Stenlund H, Svanborg E. Snoring, pregnancy-induced hypertension, and growth retardation of the fetus. Chest. 2000 Jan;117(1):137-41.

17. Flenady V, Koopmans L, Middleton P, Froen JF, Smith GC, Gibbons K, et al. Major risk factors for stillbirth in high-income countries: a systematic review and meta-analysis. Lancet. 2011 Apr 16;377(9774):1331-40.

18. Warland J, Mitchell EA. A triple risk model for unexplained late stillbirth. BMC pregnancy and childbirth. 2014 Apr 14;14:142.

19. Stewart LA, Clarke M, Rovers M, Riley RD, Simmonds M, Stewart G, et al. Preferred Reporting Items for Systematic Review and Meta-Analyses of individual participant data: the PRISMA-IPD Statement. Jama. 2015 Apr 28;313(16):1657-65.

For peer review only

For peer review only - http://bmjopen.bmj.com/site/about/guidelines.xhtml

20. Riley RD, Lambert PC, Abo-Zaid G. Meta-analysis of individual participant data: rationale, conduct, and reporting. Bmj. 2010 Feb 05;340:c221.

21. Debray TP, Riley RD, Rovers MM, Reitsma JB, Moons KG, Cochrane IPDM-aMg. Individual participant data (IPD) meta-analyses of diagnostic and prognostic modeling studies: guidance on their use. PLoS medicine. 2015 Oct;12(10):e1001886.

22. Ahmed I, Debray TP, Moons KG, Riley RD. Developing and validating risk prediction models in an individual participant data meta-analysis. BMC medical research methodology. 2014 Jan 08;14:3.

23. Tudur Smith C, Hopkins C, Sydes MR, Woolfall K, Clarke M, Murray G, et al. How should individual participant data (IPD) from publicly funded clinical trials be shared? BMC medicine. 2015 Dec 17;13:298.

24. WHO. Maternal, newborn, child and adolescent health: Data, statistics and epidemiology: WHO; [cited 2016 21 November 2016]. Available from: http://www.who.int/maternal_child_adolescent/epidemiology/stillbirth/en/.

25. Morgan R. The ROBINS-E tool (Risk Of Bias In Non-randomized Studies - of Exposures): University of Bristol; 2017 [cited 2017 20th October]. Available from: http://www.bristol.ac.uk/population-health-sciences/centres/cresyda/barr/riskofbias/robins-e/.

For peer review only - http://bmjopen.bmj.com/site/about/guidelines.xhtml

Appendix 1:

Search strategy for the Collaborative IPD of Sleep and Stillbirth (Cribss) study

Databases or search engines that will be used

A search of the databases: MEDLINE, EMBASE, LILACS, Web of Science, OpenGrey, and Google Scholar, will be conducted, for the purpose of locating published research about an association between maternal sleep position and late pregnancy stillbirth. We will also access WHO International Clinical Trials Registry Platform to identify any ongoing and registered trials. Proceedings from International Stillbirth Alliance (ISA) annual conferences and The International Society for the Study and Prevention of Perinatal and Infant Death (ISPID) international conferences will be manually searched. Published perinatal conference abstracts will be identified through the above database searches. Experts in the field and the collaborative group will be asked for their knowledge of any unpublished studies.

Limits applied

To increase the likelihood of identifying all relevant studies, the reference lists of all retrieved articles will be hand searched. No language restriction will be applied.

List the search terms used

Three search terms will be used to search the databases with the article title, abstracts and body all searched. The search terms are:

- stillbirth
- fetal death
- sleep

and synonyms. The search terms will be tested to check that they effectively located the types of articles that are consistent with the inclusion criteria prior to conducting the search in all engines.

Document the search process

The following search was conducted sequentially using the search terms in MEDLINE on 20th November 2016.

Search engine		Search terms	# Retrieved:
**MEDLINE**			
MEDLINE	1	Stillbirth/	3851
MEDLINE	2	(stillbirth* or still-birth* or stillborn* or still-born*).ti,ab,kf.	13691
MEDLINE	3	Fetal Death/	24585
MEDLINE	4	((fetal or foetal or fetus or foetus) adj death*).ti,ab,kf.	8769
MEDLINE	5	((fetal or foetal or fetus or foetus) adj3 (loss or losses)).ti,ab,kf.	4804
MEDLINE	6	Perinatal Death/	860
MEDLINE	7	((perinatal or peri-natal) adj death*).ti,ab,kf.	4007
MEDLINE	8	((prenatal or pre-natal or intrauterine or intra-uterine or antepartum or ante-partum or antenatal or ante-natal) adj death*).ti,ab,kf.	2026
MEDLINE	9	or/1-8	46353
MEDLINE	10	Sleep/	46957
MEDLINE	11	((sleep or sleeping) adj (position* or practice* or posture*)).ti,ab,kf.	1354
MEDLINE	12	maternal sleep*.ti,ab,kf.	139
MEDLINE	13	or/10-12	47711
MEDLINE	14	9 and 13	23

For peer review only

Collaborative IPD Analysis of Sleep and Stillbirth

Statistical Analysis Plan Version 2

Project Title An individual participant data meta-analysis of going-to-sleep position and risk of late pregnancy stillbirth

Field of research 11402 Obstetrics and Gynaecology

Date of first version	17/08/2017
Last updated	28/01/2018

For peer review only

Table of content

1. INTRODUCTION AND OVERVIEW ... 1

1.1. STUDY OVERVIEW ... 1

1.2. OBJECTIVES ... 1

1.3. ELIGIBILITY CRITERIA ... 1

1.4. DEFINITION OF PRIMARY OUTCOME ... 2

1.5. DEFINITION OF SECONDARY OUTCOME ... 3

1.6. DEFINITION OF PRIMARY EXPOSURE ... 3

1.7. DEFINITION OF OTHER VARIABLES ... 4

1.7.1. Potential confounders for main questions ... 4

1.7.2. Additional variables for secondary questions ... 7

2. STATISTICAL ANALYSIS PLAN ... 8

2.1. ANALYSIS POPULATION ... 8

2.2. SAMPLE SIZE ... 8

2.3. DESCRIPTIVE STATISTICS ... 8

2.4. ANALYSIS FOR MAIN QUESTIONS ... 8

2.5. ANALYSIS FOR SECONDARY QUESTIONS ... 9

REFERENCES ... 10

For peer review only

1. Introduction and Overview

1.1. Study overview

Accumulating evidence has shown an association between maternal supine sleep position and stillbirth in late pregnancy. Advising women not to go to sleep on their back can potentially reduce late stillbirth rate by 3.7%-10% [1-3]. However, the association between maternal right side sleep position and stillbirth is inconsistent across studies. Furthermore, individual studies are underpowered to investigate interactions between maternal sleep position and fetal vulnerability, which is potentially important for producing clear and tailored public health messages on safe going-to-sleep position. We aim to use individual participant data (IPD) from existing studies to assess whether right-side and supine going-to-sleep positions are independent risk factors for late stillbirth and test the interaction between sleep position and indicators of fetal vulnerability.

1.2. Objectives

The main questions to be addressed:

1. Is maternal going-to-sleep position associated with late stillbirth?
2. Are indicators of fetal vulnerability, including: maternal obesity, SGA, maternal smoking, maternal second-hand tobacco exposure, substance use, alcohol consumption, maternal medical conditions (including pre-existing hypertension and diabetes), and maternal perception of fetal movements associated with late stillbirth, and does maternal going-to-sleep position interact with indicators of fetal vulnerability to influence the risk of late stillbirth? Does birthweight centile interact with maternal going-to-sleep position to influence the risk of late stillbirth?

Secondary questions to be addressed by the first cycle of Cribss IPD meta-analysis are:

1. Is sleep disturbed breathing associated with late stillbirth? Is (are) going-to-sleep position(s) associated with greater risk of late stillbirth in women with sleep

For peer review only - http://bmjopen.bmj.com/site/about/guidelines.xhtml

disturbed breathing? Is sleep disturbed breathing a moderator for sleep position in relation to late stillbirth?

2. Are factors that may influence vena caval compression (eg, long sleep duration, sleeping during the day, restless legs,) associated with risk of late stillbirth? Do these factors interact with going-to-sleep position?
3. Do women who report they received advice about sleep position have lower risk of late stillbirth compared with women who did not receive such advice?
4. Do women who report they received advice about awareness of fetal movements have a lower risk of late stillbirth than women who did not receive such advice?

1.3. Eligibility criteria

Study inclusion criteria (regardless of whether the study is published or unpublished):

1. Case-control and prospective cohort studies which collected:
   - Maternal going-to-sleep position during pregnancy and
   - Pregnancy outcome that included stillbirth and
   - Aimed to recruit controls with an on-going pregnancy at similar gestation to the cases
2. Randomised controlled trials which collected:
   - Maternal going-to-sleep position during pregnancy and
   - Pregnancy outcome data that included stillbirth

Participant level exclusion criteria:

- Multiple pregnancy in the third trimester
- Major congenital abnormality at study entry or major congenital abnormality as a cause of death found post study entry or post-randomisation in randomised controlled trials

For peer review only - http://bmjopen.bmj.com/site/about/guidelines.xhtml

- Gestation less than 28 weeks when last sleep position data during pregnancy was collected
- Termination of pregnancy at greater than or equal to 28 weeks
- Received study intervention that might have an impact on going-to-sleep position.

1.4. Definition of Primary Outcome

Late stillbirth

We will use the definition recommended by World Health Organisation (WHO) for international comparison: “a baby born with no signs of life at or after 28 weeks' gestation”[4]. Intrapartum stillbirth will be included in the analysis with the rationale that supine going-to-sleep position may result in a vulnerable baby that is unable to tolerate labour.

1.5. Definition of Secondary Outcome

Small for Gestational Age (SGA)

SGA will be defined using (1) the definition in each study, (2) Customised centiles [5, 6], (3) WHO or uniform population standards, and (4) INTERGROWTH-21st. For primary analysis, SGA is defined as birthweight less than the 10th customized birth centile. Mother estimated date of stillbirth (before or on the same day of the diagnosis of stillbirth) will be used to calculate the gestation for SGA. If estimated date of stillbirth is unavailable, date of diagnosis of stillbirth will be used. If date of diagnosis is unavailable, baby date of birth will be used.

1.6. Definition of Primary Exposure

Maternal going-to-sleep position

Going-to-sleep position for the primary analysis is the information collected closest to time of interview on going-to-sleep position but within the ‘last two weeks’ prior to interview in controls and before stillbirth in cases. It has been shown in previous studies, that last night going-to-sleep position is highly correlated to the usual going-to-sleep position within the last two weeks. Going-to-sleep position will be categorised as left side, right side, variable sides, supine, prone, and propped up. Left side will be used as the reference group during

For peer review only - http://bmjopen.bmj.com/site/about/guidelines.xhtml

the analysis. Depending on the similarity of the risk estimates, going-to-sleep position may be further merged to fewer groups such as supine vs non-supine groups in the analysis of interaction.

1.7. Definition of Other Variables

1.7.1. Potential confounders for main questions

Maternal age

Maternal age is defined as the age at the time of interview for controls and the age at the time of stillbirth for cases. Maternal age should be calculated by date of birth where possible. Self-reported age at the interview will be used if maternal date of birth or date of interview is unavailable. The relationship between maternal age and stillbirth will be explored using a generalized additive model (GAM). Depending on the linearity of the relationship, maternal age will be further explored as either a continuous variable or a categorical variable.

Maternal ethnicity

Maternal ethnicity is defined as self-reported ethnicity. Maternal ethnicity will be assessed 1) per original study protocol, eg: using prioritization in New Zealand studies [7]. 2) by IPD agreed standardization rule and will be categorised as White (including NZ and Australia European, British, Irish and Gypsy, and other Europeans), Black (including British Black, African, and Caribbean), South Asian (including Indian, Pakistani, Bangladeshi, Sri Lankan, Nepali, Bhutanese, Afghan and Maldivian), South East and East Asian (including Chinese, Japanese, Korean, Vietnamese, Malaysian, and Indonesian), Maori, Pacific Islanders, and others. If the number in some pre-defined group is insufficient for analysis, ethnicity may be further aggregated into fewer groups such as white and non-white.

Maternal parity

Parity is defined as number of previous births after 24 weeks gestation. Maternal parity will be assessed by IPD agreed standardization rule (births after 24 weeks gestation). Maternal parity will be initially explored as six groups: 0, 1, 2, 3, 4, and 5 or more. Depending on the similarity of the risk estimate, maternal parity will be further merged to fewer groups where appropriate.

For peer review only - http://bmjopen.bmj.com/site/about/guidelines.xhtml

Maternal education level

Maternal education level will be used as a surrogate for social deprivation and is defined as the highest education the participant has completed at the time of interview. Maternal education level will be explored as five groups: 1. Primary; 2. Secondary; 3. Non-university trade education (vocational training); 4. University; 5. Post-graduate degree. Depending on the similarity of the risk estimates, maternal education level will be further merged to fewer groups where appropriate.

Marital status

Marital status will be categorised as single (including never married, divorced, widowed and separated), co-habiting (including de facto) or married (including civil partnership). Depending on the data availability, marital status can be also categorised as single or in relationship (cohabiting and married).

Maternal BMI

Maternal BMI is defined as the earliest collected weight during pregnancy or before pregnancy (kg) divided by squared maternal height (m). Where maternal weight or maternal height is unavailable, the earliest BMI recorded during or before pregnancy will be used. The proportion of participants with their earliest weight measurement recorded during the first trimester will be calculated.

The relationship between maternal BMI and stillbirth (eg: any dose –response relation) will be explored using GAM. Depending on the linearity of the relationship, maternal age will be further explored as either a continuous variable or a categorical variable.

Maternal obesity

Maternal earliest BMI during pregnancy equal to or greater than 30 is considered as obese. Maternal obesity will be explored as an indication of fetal vulnerability.

Maternal smoking

Maternal smoking status will be explored as three groups: current smoker (including those who stopped in 2nd, or 3rd trimester), used to smoke but stopped within the 1st trimester, and never smoked. Depending on the similarity of the risk estimate, maternal smoking status may be merged to two groups in the final analysis.

For peer review only - http://bmjopen.bmj.com/site/about/guidelines.xhtml

Environmental tobacco exposure

Environmental tobacco exposure is defined as living with a smoker (including partner or any other person living in the same household) anytime during pregnancy.

Substance use

Maternal substance use will be defined as: 1) any use of recreational drugs during any stage in pregnancy, 2) marijuana use in the first three months during pregnancy, 3) marijuana use in the last month during pregnancy, and 4) marijuana in the last week before interview / stillbirth.

Alcohol consumption

Maternal alcohol exposure will be assessed by the largest number of standard drinks on one occasion during any phase of pregnancy. Maternal alcohol exposure will be also assessed by the average standard drinks per week in the month before interview for controls and before stillbirth for cases. Average standard drinks per week will be coded as 'less than 1', '1 to 2', '3 to 4', and '5 or more'. Depending on the similarity of the risk estimates, maternal alcohol exposure may be merged to fewer groups in the final analysis.

Maternal medical conditions (eg: diabetes, hypertension)

Maternal medical conditions will be defined as per original study using local clinical diagnoses as most of the information required to standardise diagnostic criteria across studies is unavailable. However, local diagnostic criteria will be compared if any medical condition is found to be an effect modifier.

Maternal perception of fetal movements

Maternal perception of fetal movements will use changes in fetal movement frequency within the last two weeks for the main questions. Changes in fetal movement frequency will be defined as 1) increased, 2) decreased and 3) same. Particularly, unknown changes will be categorised as same.

Getting up to go to toilet

This is defined as maternal self-reported number of times getting up to go to the toilet on the last night, or if last night information is unavailable, self-reported average over the 'last two weeks' prior to interview in controls and before stillbirth in cases.

For peer review only - http://bmjopen.bmj.com/site/about/guidelines.xhtml

For peer review only

Sleep duration

This is defined as self-estimated sleep duration for the last night, or if last night information is unavailable, self-reported average over the 'last two weeks' prior to interview in controls and before stillbirth in cases.

Daytime napping

Daytime napping is defined as self-reported frequency of sleep during day time per week in the most recent available time frame during pregnancy.

Birthweight centile

Birthweight centile will be calculated using (1) the definition in each study, (2) Customised centiles [5, 6], (3) WHO or uniform population standards, and (4) INTERGROWTH-21st.

For peer review only

1.7.2. Additional variables for secondary questions

Sleep disturbed breathing

Questions adopted from the Berlin questionnaire for obstructive sleep apnea will be used to investigate sleep disturbed breathing in this study [8]. The following information will be evaluated: (1) awareness of snoring or not, (2) if the snoring bothering other people, (3) snoring volume, (4) stopping breathing during the sleep, (5) coughing or choking during sleep, (6) daytime sleepiness, (7) the likelihood of doing off at various occasions including sitting while reading, watching TV, sitting in public, sitting in a car, lying down for rest in the afternoon, sitting while talking, after lunch, and in a car when stopping at the traffic light.

Restless legs syndrome

Restless legs syndrome is defined as regular jerking movements of arms or legs during sleep during any phase of the pregnancy.

Advice about sleep position

Advice about sleep position will be assessed as binary data- received advice on sleep position or not during pregnancy. The sources and the content of the advice will be evaluated further according to the data availability.

Advice about fetal movement

For peer review only - http://bmjopen.bmj.com/site/about/guidelines.xhtml

Advice about fetal movement will be assessed as binary data: 1)received advice on fetal movement 2) did not receive advice or 3) cannot recall advice during pregnancy. The sources and the content of the advice will be evaluated further according to the data availability.

2. Statistical Analysis Plan

2.1. Analysis population

Participants who meet the above inclusion criteria (see 1.3) will be included in the analysis. Participants with missing data for variables to be included in multivariable model will not have those data-points imputed.

2.2. Sample size

We anticipate that 700 cases and 1800 controls will be included in the primary analysis. This sample size will have 80% power to detect an odds ratio of 1.86 for a factor with 3% prevalence in controls, and an odds ratio of 1.47 for a factor with 10% prevalence in controls, and an odds ratio of 1.35 for a factor with 20% prevalence in controls.

2.3. Descriptive statistics

Descriptive statistics for exposure and confounders will be presented in tables by cases and controls. All data will be explored for missing data and checked for distribution.

Continuous variables: GAM will be used to explore their relationship between continuous variables and the main outcome (late stillbirth). If the relationship is linear, the variable will be presented as mean and standard deviation, or median and quartiles when considered appropriate, and will be fitted in the logistic model as continuous variables. If the relationship is not linear, the variable will be categorised or fitted with non-linear terms.

Categorical variables: categorical variables will be described in terms of prevalence (frequency) and percentages of the number of participants examined.

2.4. Analysis for main questions

An individual participant data (IPD) analysis will be performed. A one stage approach to analysis will be taken so that the individual participant data from all eligible studies are included in a single model. Logistic regression models will be used for the binary outcome

For peer review only - http://bmjopen.bmj.com/site/about/guidelines.xhtml

(late stillbirth). A fixed study effect and a study site effect will be included in the model specification as strata. Univariable analysis will be performed to evaluate the association between sleep position and late stillbirth risk. The interaction between sleep position and factors indicating a vulnerable pregnancy will be assessed in bi-variable models. A multivariable model will be developed incorporating previously reported confounders and any significant interaction terms, once it has been established what cofounders can be controlled for consistently across studies. Estimate of risk will be reported as odds ratio and 95% confidence intervals.

Statistical analyses will be performed using SAS (SAS Institute Inc., Cary NC USA). If an important confounder is not available for one or more studies, sensitivity analysis will be conducted, with and without these studies, to compare risk estimates. For missing data in each individual study, no imputation will be carried out.

2.5. Analysis for secondary questions

Secondary questions investigating other exposures and the risk of stillbirth will be first analysed by each study, and then the merged data set will be analysed through a one stage approach. The rationale to first explore each study independently is because these questions have never been explored before and it is important to see if there is any exposure outcome effect in each individual study. Logistic regression models will be used for the binary outcome (late stillbirth). A fixed study effect and a study site effect will be included in models as strata. Univariable analysis will be performed to evaluate the association between exposures (data indicating sleep disturbed breathing, indications of compression of vena cava, and advice of sleep position and fetal movement) and late stillbirth risk. The interaction between secondary exposures and sleep position will be explored in bi-variable models. A multivariable model will be developed incorporating appropriate confounders/interaction. If an important confounder is not available for one or more studies, sensitivity analysis will be conducted, with and without these studies, to compare risk estimates. Estimate of risk will be reported as odds ratio and 95% confidence intervals.

Statistical analyses will be performed using SAS (SAS Institute Inc., Cary NC USA).

For peer review only - http://bmjopen.bmj.com/site/about/guidelines.xhtml

References

1. McCowan LME, Thompson JMD, Cronin RS, Li M, Stacey T, Stone PR, et al. Going to sleep in the supine position is a modifiable risk factor for late pregnancy stillbirth; Findings from the New Zealand multicentre stillbirth case-control study. PloS one. 2017;12(6):e0179396.

2. Platts J, Mitchell EA, Stacey T, Martin BL, Roberts D, McCowan L, et al. The Midland and North of England Stillbirth Study (MiNESS). BMC pregnancy and childbirth. 2014;14:171.

3. Gordon A, Raynes-Greenow C, Bond D, Morris J, Rawlinson W, Jeffery H. Sleep position, fetal growth restriction, and late-pregnancy stillbirth: the Sydney stillbirth study. Obstetrics and gynecology. 2015 Feb;125(2):347-55.

4. WHO. Maternal, newborn, child and adolescent health: Data, statistics and epidemiology: WHO; [cited 2016 21 November 2016]. Available from: http://www.who.int/maternal_child_adolescent/epidemiology/stillbirth/en/.

5. Gardosi J, Chang A, Kalyan B, Sahota D, Symonds EM. Customised antenatal growth charts. Lancet. 1992 Feb 01;339(8788):283-7.

6. McCowan L, Stewart AW, Francis A, Gardosi J. A customised birthweight centile calculator developed for a New Zealand population. The Australian & New Zealand journal of obstetrics & gynaecology. 2004 Oct;44(5):428-31.

7. Minitry of Health. Ethnicity Data Protocols for the Health and Disability Sector. Wellington: Ministry of Health2004. Available from: http://www.health.govt.nz/publication/ethnicity-data-protocols-health-and-disability-sector

8. Netzer NC, Stoohs RA, Netzer CM, Clark K, Strohl KP. Using the Berlin Questionnaire to identify patients at risk for the sleep apnea syndrome. Annals of internal medicine. 1999 Oct 05;131(7):485-91.

For peer review only - http://bmjopen.bmj.com/site/about/guidelines.xhtml

PRISMA-P (Preferred Reporting Items for Systematic review and Meta-Analysis Protocols) 2015 checklist: recommended items to address in a systematic review protocol*

Section and topic	Item No	Checklist item	Check
**ADMINISTRATIVE INFORMATION**			
Title:			
Identification	1a	Identify the report as a protocol of a systematic review	Yes, P1, line 2
Update	1b	If the protocol is for an update of a previous systematic review, identify as such	na
Registration	2	If registered, provide the name of the registry (such as PROSPERO) and registration number	Yes, P3, line 60
Authors:			
Contact	3a	Provide name, institutional affiliation, e-mail address of all protocol authors; provide physical mailing address of corresponding author	Yes, P2, line 4-36
Contributions	3b	Describe contributions of protocol authors and identify the guarantor of the review	Yes, P17, line 346-350
Amendments	4	If the protocol represents an amendment of a previously completed or published protocol, identify as such and list changes; otherwise, state plan for documenting important protocol amendments	na
Support:			
Sources	5a	Indicate sources of financial or other support for the review	Yes, P17, line 353
Sponsor	5b	Provide name for the review funder and/or sponsor	Yes, P17, line 353
Role of sponsor or funder	5c	Describe roles of funder(s), sponsor(s), and/or institution(s), if any, in developing the protocol	Yes, P17, line 353
**INTRODUCTION**			
Rationale	6	Describe the rationale for the review in the context of what is already known	Yes, P5, line 80-166
Objectives	7	Provide an explicit statement of the question(s) the review will address with reference to participants, interventions, comparators, and outcomes (PICO)	Yes, P8, line 163
**METHODS**			
Eligibility criteria	8	Specify the study characteristics (such as PICO, study design, setting, time frame) and report characteristics (such as years considered, language, publication status) to be used as criteria for eligibility for the review	Yes, P9, line 195
Information sources	9	Describe all intended information sources (such as electronic databases, contact with study authors, trial registers or other grey literature sources) with planned dates of coverage	Yes, P9-10, line 214

For peer review only

Search strategy	10	Present draft of search strategy to be used for at least one electronic database, including planned limits, such that it could be repeated	Yes, P9-10, line 214 and appendix 1
Study records:			
Data management	11a	Describe the mechanism(s) that will be used to manage records and data throughout the review	Yes, P11, line 247-277
Selection process	11b	State the process that will be used for selecting studies (such as two independent reviewers) through each phase of the review (that is, screening, eligibility and inclusion in meta-analysis)	Yes, P10, line 236
Data collection process	11c	Describe planned method of extracting data from reports (such as piloting forms, done independently, in duplicate), any processes for obtaining and confirming data from investigators	Yes, P11, line 247-277
Data items	12	List and define all variables for which data will be sought (such as PICO items, funding sources), any pre-planned data assumptions and simplifications	Yes, line 279-283 (table 1)
Outcomes and prioritization	13	List and define all outcomes for which data will be sought, including prioritization of main and additional outcomes, with rationale	Yes, P14-15, line 284
Risk of bias in individual studies	14	Describe anticipated methods for assessing risk of bias of individual studies, including whether this will be done at the outcome or study level, or both; state how this information will be used in data synthesis	Yes, P15, line 290
Data synthesis	15a	Describe criteria under which study data will be quantitatively synthesised	Yes, P15, line 296
	15b	If data are appropriate for quantitative synthesis, describe planned summary measures, methods of handling data and methods of combining data from studies, including any planned exploration of consistency (such as I^2, Kendall's τ)	Yes, P15, line 296
	15c	Describe any proposed additional analyses (such as sensitivity or subgroup analyses, meta-regression)	Yes, P16, line 315
	15d	If quantitative synthesis is not appropriate, describe the type of summary planned	na
Meta-bias(es)	16	Specify any planned assessment of meta-bias(es) (such as publication bias across studies, selective reporting within studies)	Yes, P16, line 315
Confidence in cumulative evidence	17	Describe how the strength of the body of evidence will be assessed (such as GRADE)	na

*** It is strongly recommended that this checklist be read in conjunction with the PRISMA-P Explanation and Elaboration (cite when available) for important clarification on the items. Amendments to a review protocol should be tracked and dated. The copyright for PRISMA-P (including checklist) is held by the PRISMA-P Group and is distributed under a Creative Commons Attribution Licence 4.0.**

From: Shamseer L, Moher D, Clarke M, Ghersi D, Liberati A, Petticrew M, Shekelle P, Stewart L, PRISMA-P Group. Preferred reporting items for systematic review and meta-analysis protocols (PRISMA-P) 2015: elaboration and explanation. BMJ. 2015 Jan 2;349(jan02 1):g7647.

For peer review only
